# Engineering of *Streptomyces lividans* for heterologous expression of secondary metabolite gene clusters

**DOI:** 10.1186/s12934-020-1277-8

**Published:** 2020-01-09

**Authors:** Yousra Ahmed, Yuriy Rebets, Marta Rodríguez Estévez, Josef Zapp, Maksym Myronovskyi, Andriy Luzhetskyy

**Affiliations:** 1 Pharmazeutische Biotechnologie, Universität des Saarlandes, Saarbrücken, Germany; 2 Pharmazeutische Biologie, Universität des Saarlandes, Saarbrücken, Germany; 3 Helmholtz-Institut für Pharmazeutische Forschung Saarland, Saarbrücken, Germany

**Keywords:** Heterologous expression, *Streptomyces*, Natural product, Heterologous host, Gene cluster

## Abstract

**Background:**

Heterologous expression of secondary metabolite gene clusters is used to achieve increased production of desired compounds, activate cryptic gene clusters, manipulate clusters from genetically unamenable strains, obtain natural products from uncultivable species, create new unnatural pathways, etc. Several *Streptomyces* species are genetically engineered for use as hosts for heterologous expression of gene clusters. *S. lividans* TK24 is one of the most studied and genetically tractable actinobacteria, which remain untapped. It was therefore important to generate *S. lividans* chassis strains with clean metabolic backgrounds.

**Results:**

In this study, we generated a set of *S. lividans* chassis strains by deleting endogenous gene clusters and introducing additional φC31 *attB* loci for site-specific integration of foreign DNA. In addition to the simplified metabolic background, the engineered *S. lividans* strains had better growth characteristics than the parental strain in liquid production medium. The utility of the developed strains was validated by expressing four secondary metabolite gene clusters responsible for the production of different classes of natural products. Engineered strains were found to be superior to the parental strain in production of heterologous natural products. Furthermore, *S. lividans*-based strains were better producers of amino acid-based natural products than other tested common hosts. Expression of a *Streptomyces albus* subsp*. chlorinus* NRRL B-24108 genomic library in the modified *S. lividans* ΔYA9 and *S. albus* Del14 strains resulted in the production of 7 potentially new compounds, only one of which was produced in both strains.

**Conclusion:**

The constructed *S. lividans*-based strains are a great complement to the panel of heterologous hosts for actinobacterial secondary metabolite gene expression. The expansion of the number of such engineered strains will contribute to an increased success rate in isolation of new natural products originating from the expression of genomic and metagenomic libraries, thus raising the chance to obtain novel biologically active compounds.

## Background

*Streptomyces* is considered one of the most explored genera of *Actinobacteria*. These bacteria produce a large number of pharmaceutically important compounds as a part of their secondary metabolism [1, 2]. Secondary metabolite biosynthesis genes are typically grouped into clusters that include structural genes coding for biosynthetic enzymes as well as regulatory and resistance/transport genes. Such an organization simplifies the identification of biosynthetic genes and facilitates their cloning and expression in heterologous hosts. This heterologous expression is especially useful since it allows fast and simple manipulations with gene clusters of interest, which otherwise can be difficult to handle in natural producers since many actinobacteria are poorly genetically tractable. Heterologous expression is often used to activate silent gene clusters [3, 4], generate unnatural metabolites by combinatorial biosynthesis or mutasynthesis [5, 6], and obtain a better yield of compounds of interest [7]. To obtain the best outcome from this procedure, an appropriate expression host that is well studied and genetically amenable as well as provides a high pool of precursors and energy must be used. Therefore, several *Streptomyces* species, such as *S. coelicolor*, *S. lividans*, *S. avermitilis* and *S. albus,* are used as hosts for the expression of gene clusters cloned from actinomycetes. Originally, the native strains were employed in such experiments. However, this approach often caused difficulties in the identification of produced metabolites or interactions between expressed and endogenous pathways, resulting in aberrant product formation [8, 9]. To overcome these complications, the first modified host strain, *S. coelicolor* CH999, deficient in the production of internal natural products was constructed [10]. This strain was generated by deleting the (*act*) actinorhodin biosynthetic genes and inactivating undecylprodigiosin (*red*) gene clusters and was primarily used to study the functional peculiarities of the type II PKS system. With the advent of the genomic era in actinobacteria research, the idea of “clean” chassis strains became feasible. As a result, several improved hosts were constructed with a simplified metabolic background, an enhanced supply of precursors and an increased productivity of desired exogenous secondary metabolites. Komatsu and Ikeda generated genome-minimized derivatives of *S. avermitilis*, SUKA5, SUKA17 and SUKA22 [11]. The *S. avermitilis* SUKA5 strain has a deletion of the oligomycin biosynthetic gene cluster as well as in the left subtelomeric region covering avermectin and filipin biosynthetic gene clusters. SUKA17 and SUKA22 are isogenic strains that contain additional deletions of pentalenolactone-, geosmin- and carotenoid-encoding gene clusters. Different heterologous secondary metabolites were successfully produced in engineered *S. avermitilis* strains, including the aminoglycoside streptomycin, the β-lactam cephamycin C and the macrocyclic compound pladienolide [11]. Gomez-Escribano and Bibb developed the *S. coelicolor* M1152 and M1154 strains with deletion of four internal gene clusters (*act*, *red*, *cpk*, encoding coelimycin; and *cda,* encoding a calcium-dependent antibiotic). In addition, M1152 carries an *rpoB* (rifampicin resistance) mutation, and M1154 has *rpoB* and *rpsL* (streptomycin resistance) mutations [12]. These mutations were reported to enhance the production of secondary metabolites in actinobacteria due to increased RNA polymerase promoter affinity (*rpoB*) and induction of protein synthesis in the stationary growth phase (*rpsL*) [13, 14]. *S. coelicolor* strains have been widely used to express different types of secondary metabolite biosynthetic gene clusters (reviewed in [5]). The *S. albus* J1074 derivative *S. albus* Del14 with deletion of 15 endogenous gene clusters is another engineered host strain with a “clean” genetic and metabolic background for secondary metabolite gene cluster expression [15]. *S. albus* Del14 was successfully used to activate the cryptic type I PKS gene cluster from the metagenomic library, resulting in the production of pyridinopyrone. Furthermore, two other cryptic clusters from *Frankia alni* ACN14a and *Frankia* sp. CcI3 were successfully expressed in *S. albus* Del14, leading to the production of salicylic acid, fradiomycin (from *Frankia alni* ACN14a), bhimamycin A and aloesaponarin II (from *Frankia* sp. CcI3) [15].

*Streptomyces lividans* is closely related to *S. coelicolor,* and both are well-characterized actinobacteria species. *S. lividans* TK24 accepts methylated DNA, has low endogenous protease activity this and also contains a streptomycin-resistant mutation improving production of secondary metabolites. It makes this strain a preferable host for heterologous expression of secondary metabolite gene clusters and production of secreted recombinant proteins [13, 16–20]. Ziermann and co-authors generated two hosts, *S. lividans* K4-114 and K4-155, by removing the entire actinorhodin gene cluster from the chromosome of *S. lividans* TK24 [21]. The expression of genes for the biosynthesis of erythromycin precursor 6-deoxyerythronolide *B (*6-dEB) in *S. lividans* K4-114 and K4-155 and *S. coelicolor* CH999 resulted in approximately the same level of compound accumulation. Similarly, a *S. lividans*-based host with a deletion of the *act* and *red* gene clusters was successfully used to produce granaticin [22]. Recently, the heterologous production of mithramycin in *S. lividans* was reported to be significantly improved by deleting the *act*, *red* and *cda* gene clusters [23]. However, no deep engineering such as in the case of *S. albus* Del14 or *S. avermitilis* SUKA strains was performed with *S. lividans* TK24 to generate the completely “clean” strain.

The success of the engineered *Streptomyces* strains prompted us to generate a new *S. lividans*-based host with a clean metabolic profile and thus simplified the detection and purification of new compounds. Herein, we report the construction of optimized *S. lividans* host strains by removing 11 endogenous secondary metabolite gene clusters, accounting for 228.5 kb of the chromosome. Further modification of the strains to improve the production level was performed by introducing additional integration sites (*attB* sites) for φC31-based vectors.

## Results

### Transcriptome-based identification of actively expressed secondary metabolite gene clusters

The genome of *S. lividans* TK24 was sequenced, and twenty-five gene clusters potentially involved in the biosynthesis of secondary metabolites were identified (Additional file 1: Table S1) [24]. For a long time, the only secondary metabolites produced by the strain, which are produced only under certain laboratory conditions, were considered to be actinorhodin and undecylprodigiosin, and the remaining gene clusters were thought to be silent. To estimate the transcription of secondary metabolite genes, we performed an analysis of RNAseq data from a previously described dataset obtained for *S. lividans* TK24 cultivated in minimal medium in a mini-bioreactor [25]. The transcription of secondary metabolites genes was calculated as the average RPKM (reads per kilobase per million mapped reads) of all genes within a particular cluster identified by antiSMASH with the ClusterFinder algorithm for cluster border prediction (Additional file 1: Table S1). We found that the majority of secondary metabolites gene clusters were transcriptionally active during the stationary phase of growth. The most highly expressed gene clusters are responsible for the biosynthesis of putative rhizobactin-like siderophores (number 11, average RPKM: 918.1) [26, 27] and desferrioxamine (number 16, average RPKM: 844.3) [28]. An uncharacterized terpene cluster (number 13) was next, with an average of 803.1 RPKM; then, cluster number 21 was last, coding for biosynthesis of coelichelin with an average RPKM of 707.8. The latter compound also acts as a hydroxamate siderophore [29].

**Fig. 1:**
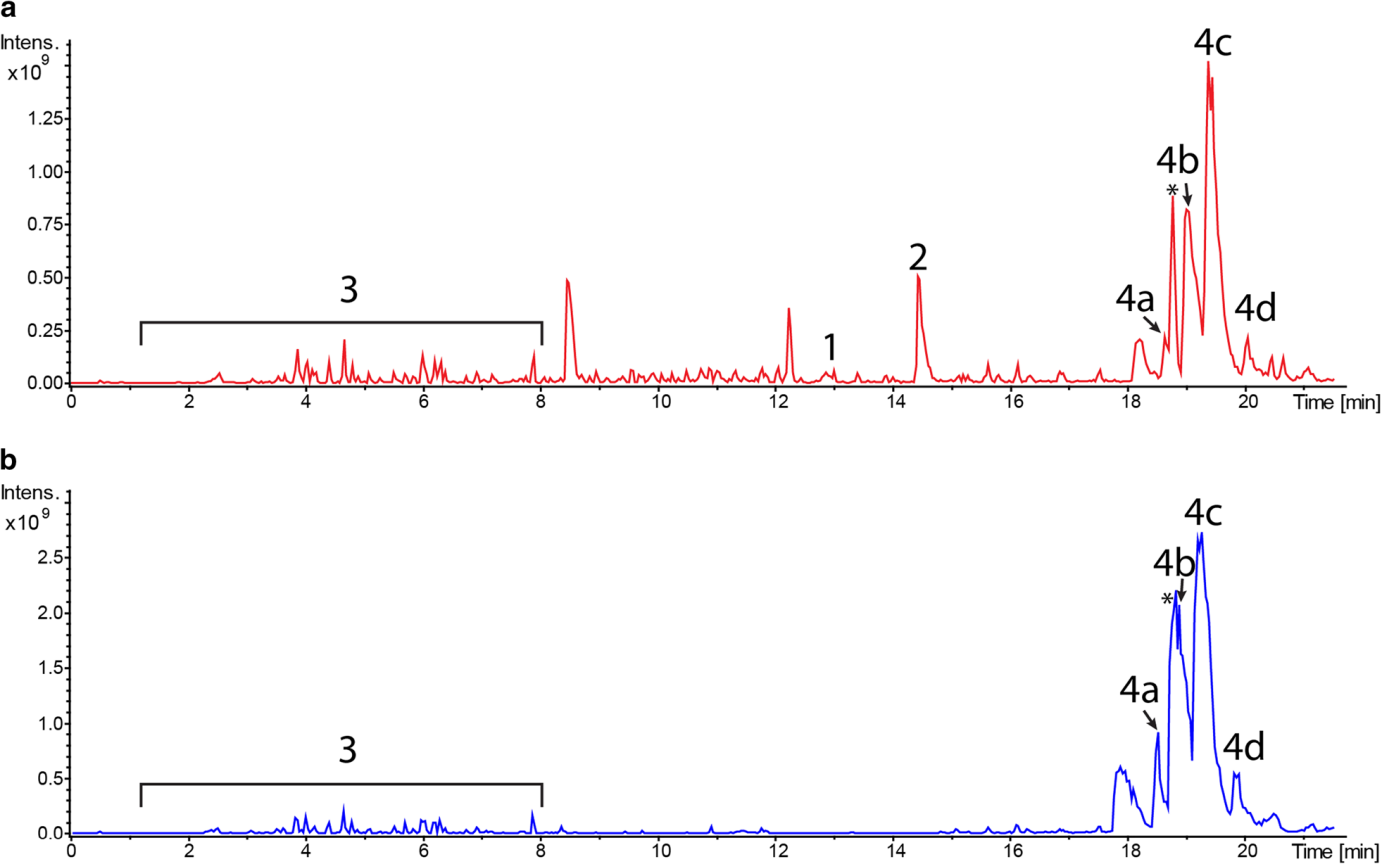
BPC chromatogram of ethyl-acetate extracts of *S. lividans* TK24 (red) and *S. lividans* ΔYA9 (blue) grown in SG medium. The identified compounds are indicated as: 1—coelibactin; 2—undecylprodigiosin; 3—media components; 4—monoglyceride lipids found in many other Streptomycetes extracts. *: unidentified contamination from the method (column bleed)

To detect the production of endogenous secondary metabolites by *S. lividans* TK24, the strain was grown in SG medium, and the metabolites were extracted and analysed by LC–MS (Fig. 1). As a result, we identified undecylprodigiosin (peak with RT of 14.4 min) and coelibactin (peak with RT of 12.9 min) in the extract of *S. lividans* TK24 (Fig. 1; Additional file 2: Fig. S1). Additionally, several compounds can be found in the extract, but we were not able to assign them to particular *S. lividans* metabolites. Actinorhodin, CDA, coelimycin P1 and other typical secondary metabolites of the strain were not found in the extract despite the observed transcriptional activity of corresponding gene clusters.

### Generation of a *S. lividans* ΔYA9 chassis strain

To simplify the metabolic profile of the strain, nine secondary metabolite biosynthesis gene clusters were deleted within the chromosome of *S. lividans* TK24, including *act*, *red*, and *cpk* clusters (Table 1). The markerless deletions were achieved by using the *S. lividans* TK24 genomic BAC library [30] and Red/ET recombination technique [31] combined with the iterative marker excision system (IMES) [32]. The recombinant BACs were constructed by replacing most of the targeted gene cluster or the core genes (in case the single BAC did not cover the entire cluster) with the IMES apramycin resistance cassette. The BAC clone was transferred into *S. lividans*, and double crossover events were selected with the help of β-glucuronidase-mediated blue-white selection [30]. To perform the sequential removal of the nine gene clusters in a single strain, deletions were intertwined with the excision of the resistance marker by expressing the φC31 phage integrase gene, leaving a 48 bp inactive scar in place of a targeted gene cluster [32]. The final engineered strain, lacking 9 gene clusters with a deletion of 178.5 kb of chromosome, was designated *S. lividans* ΔYA9. A comparison of the metabolic profiles of TK24 and ΔYA9 clearly showed an almost complete loss for ΔYA9 of the compounds detected in the extract of the parental strain, including coelibactin (**1**) and undecylprodigiosin (**2)** (Fig. 1). Only one group of closely related hydrophobic metabolites (**4a**–**d**) (Fig. 1) also found in many other streptomycetes but unidentified so far seemed to be unaffected if not even overproduced after deletion of the gene clusters. We isolated compounds **4a**–**c** and partially elucidated their structures by NMR analysis. As a result, compounds **4a**–**d** seem to be mono-substituted glyceride lipids with highly unusual unsaturated β-hydroxy fatty acids attached (Additional file 3: Fig. S2). However, due to the highly hydrophobic nature of these compounds, we were not able to purify enough chemical materials to fully solve the structure of **4a**–**d**. Although the origin of these compounds is not clear, they do not interfere with the identification of compounds resulting from heterologous gene cluster expression.

**Table 1:** Secondary metabolite gene clusters deleted within the chromosome of *S. lividans* TK24 and resulting strains

Cluster	Product	Coordinates of deleted genes	Size of the gene cluster (kb)	Size of deleted region (Kb)	Strain ID
Cluster 10	Undecylprodigiosin	09100–09185	≈ 47.65	≈ 26.5	ΔYA1
Cluster 14	Actinorhodin	12,885–12965	≈ 41.55	≈ 15.3	ΔYA2
Cluster 17	Melanin	24135–24185	≈ 9.17	≈ 8	ΔYA3
Cluster 13	Terpene	12220–12265	≈ 21.67	≈ 11.7	ΔYA4
Cluster 19	Germicidin	31668–31860	≈ 42.26	≈ 40	ΔYA5
Cluster 15	CDA	21535–21540	≈ 80.87	≈ 27	ΔYA6
Cluster 6	Coelimycin	06755–06775	≈ 79.56	≈ 20	ΔYA7
Cluster 2	Coelibactin	00885–00925	≈ 73.28	≈ 17	ΔYA8
Cluster 24	Eicosapentaenoic acid	37285–37305	≈ 53.55	≈ 13	ΔYA9
Cluster 21	Coelichelin	35395–35490	≈ 51.22	≈ 32::attB2	ΔYA10
Cluster 5	NRPS	06215–06270	≈ 48.64	≈ 18::attB3	ΔYA11

### Introduction of additional *attB* sites into the chromosome of the *S. lividans* ΔYA9 strain

Most of the gene cluster expression constructs are based on actinobacterial vectors integrating into the host chromosome. Among them, the most popular are BAC or cosmid vectors based on the φC31 and VWB actinophage integrations systems [33–35]. *S. lividans* TK24 has one major and three pseudo *attB* sites for integration of φC31-based vectors [36]. To increase the production level in our engineered strain, we decided to introduce two additional *attB* sites into *S. lividans* ΔYA9. Recombinant BACs were constructed to integrate *attB* sites in place of gene clusters 5 (an uncharacterized NRPS cluster) and 21 (coelichelin biosynthesis cluster) (see methods section). The final constructs were sequentially introduced into the *S. lividans* ΔYA9 strain by conjugation, and secondary crossover strains were selected, resulting in markerless replacement of the targeted gene clusters by *attB* sites.

We generated three different *S. lividans* strains, ΔYA9 with 9 gene clusters deleted and one native *attB* site, *S. lividans* ΔYA10 with 10 gene clusters deleted and two *attB* sites and *S. lividans* ΔYA11 with 11 gene clusters deleted and three *attB* sites (Table 1). The final strain, *S. lividans* ΔYA11, lacks 228.5 kb of chromosome (2.7%) in total.

### Growth rate and sporulation of the *S. lividans* strains

**Fig. 2:**
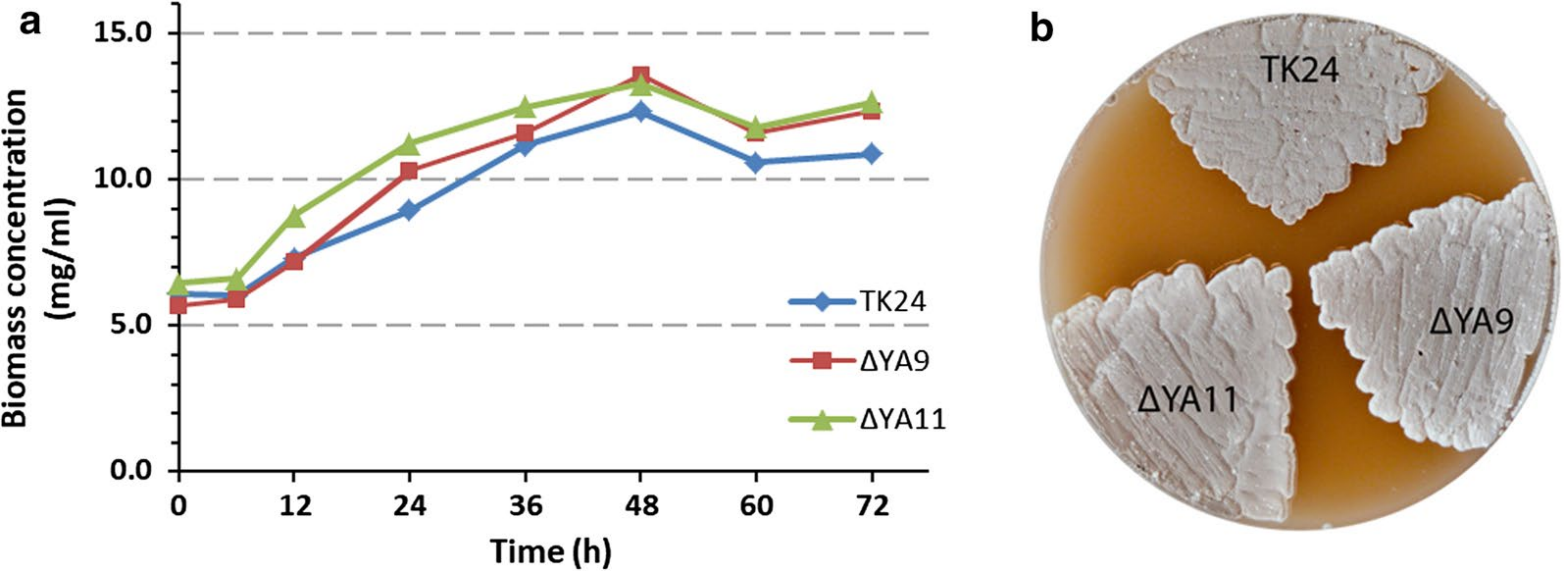
Effect of clusters deletion on fitness and growth parameters. **a** Growth curve analysis of the wild type *S. lividans* TK24 (blue) and the mutants *S. lividans* ΔYA9 (red) and *S. lividans* ΔYA11 (green) grown for 6 days in DNPM media. Samples were taken every 24 h and the wet biomass was measured. **b** Phenotype of the *S. lividans* TK24, *S. lividans* ΔYA9, and *S. lividans* ΔYA11 after 6 days growth on MS media

Actinobacterial secondary metabolites are generally accepted to have no influence on strain growth and viability except if they act as siderophores [37]. To examine the growth rate of the obtained strains, 12 mg of biomass of *S. lividans* TK24, *S. lividans* ΔYA9 and *S. lividans* ΔYA11 pre-cultures were used to inoculate 50 ml of DNPM medium. Two millilitre samples were harvested every 12 h for 3 days, and the dry biomass was weighed. The growth rates of the engineered strains were slightly higher than that of *S. lividans* TK24, resulting in an overall bigger accumulation of biomass in the case of *S. lividans* ΔYA9 and *S. lividans* ΔYA11 (Fig. 2a). Nonetheless, the three strains ended the exponential phase and entered the stationary phase of growth at similar time points, between 36 and 48 h of cultivation. The engineered strains did not show any differences in growth or morphological features on solid MS medium (Fig. 2b).

### Validation of engineered *S. lividans* strain performance

**Fig. 3:**
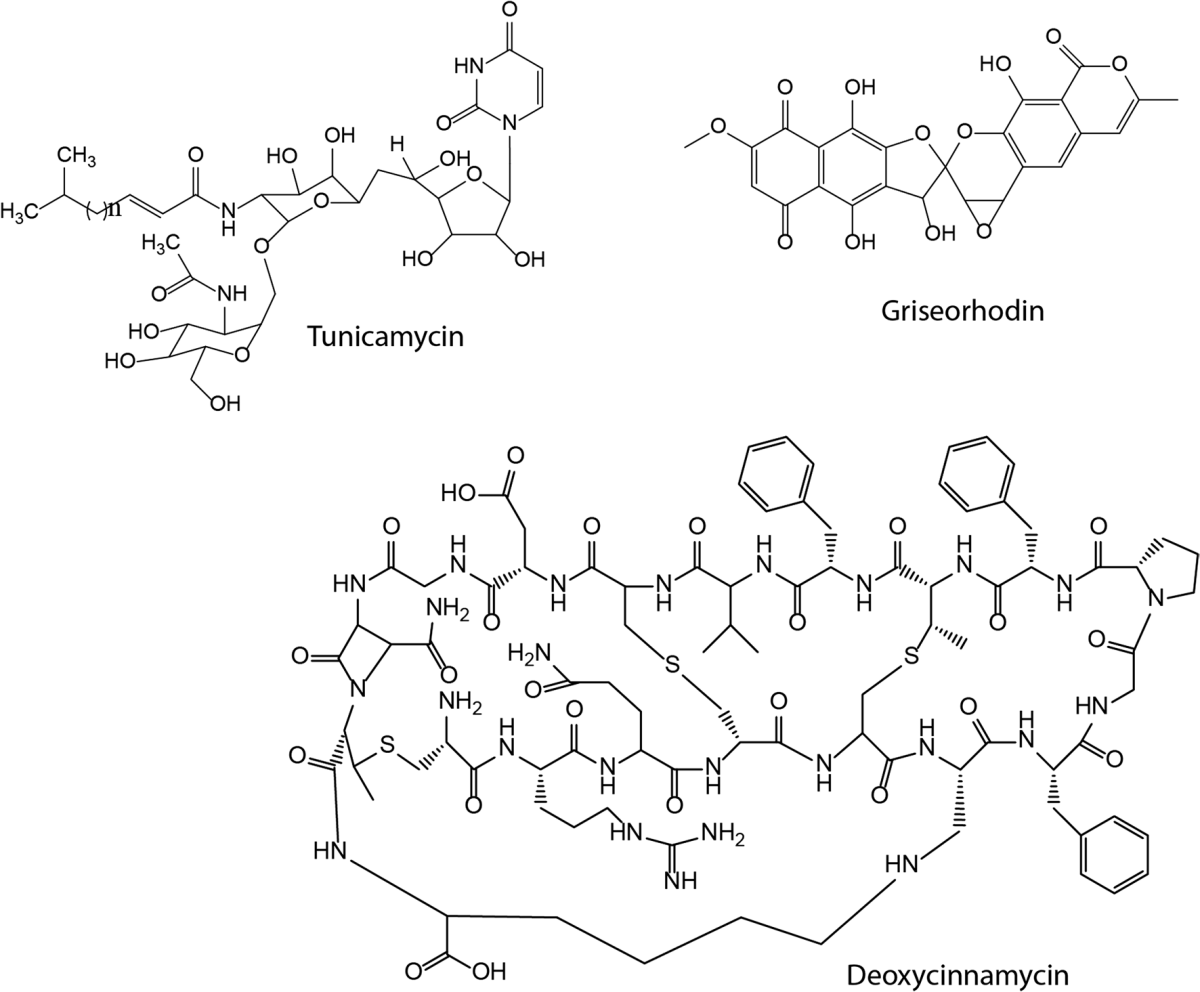
Structure of compounds described in the study

To examine the effect of gene cluster deletion and multiple copies of *attB* sites on the production level of exogenous secondary metabolites, three gene clusters representing different classes of biosynthetic pathways were chosen. Constructs carrying biosynthetic gene clusters of tunicamycin [38], griseorhodin [39], and deoxycoformycin (a derivative of cinnamycin with a deletion of a -OH group) [40] (Fig. 3) were introduced into parental and engineered *S. lividans* strains and two other engineered hosts, *S. albus* Del14 [15] and *S. coelicolor* M1154 [12]. We observed that the conjugation frequency in *S. lividans* TK24, *S. lividans* ΔYA9 and *S. albus* Del14 was similar to and higher than that in *S. lividans* ΔYA10, *S. lividans* ΔYA11 and *S. coelicolor* M1154 (data not shown). The latter strain had the lowest ex-conjugant count. The obtained recombinant strains were inoculated into two different production media: modified SG and DNPM (see materials and methods). Strains were cultivated for 6 days, and metabolites were extracted and analysed by LC–MS.

**Fig. 4:**
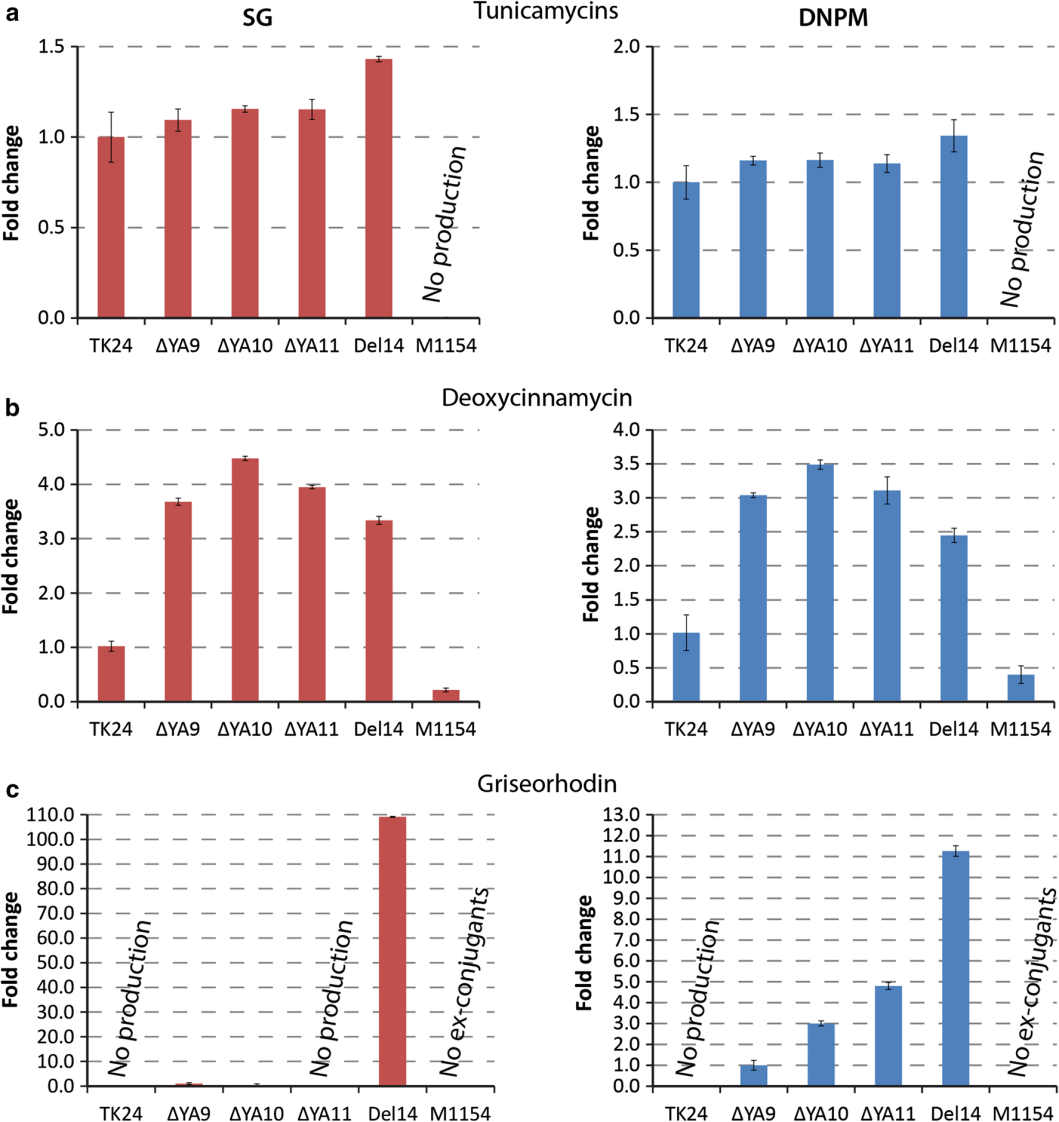
Production level of tunicamycin, griseorhodin and deoxycinnamycin in *S. lividans* TK24, *S. lividans* ΔYA9, *S. lividans* ΔYA10, *S. lividans* ΔYA11, *S. albus* Del14, *S. coelicolor* M1154. Three independent colonies from each strain were grown for 6 days in SG media (left) and DNPM (right). The production by *S. lividans* TK24 was taken as 100% in each particular experiment, except for griseorhodin, when production ΔYA9 was taken as 100%

The production level of the nucleoside antibiotic tunicamycin in engineered *S. lividans* strains was slightly higher than that in the wild-type TK24 in both media (Fig. 4a). At the same time, all engineered *S. lividans* strains produced at approximately the same level, indicating that the copy number of the gene cluster had almost no influence on the accumulation of tunicamycins. No production was observed in the case of *S. coelicolor* M1154. *S. albus* Del14 was superior to all other tested strains, superseding the best performing *S. lividans* ΔYA11 strain by approximately 20%.

Engineered *S. lividans* showed a higher production level of the lanthipeptide deoxycinnamycin than other tested strains (Fig. 4b). The production level in *S. lividans* ΔYA9 was increased more than 3.5- and threefold (in SG and DNPM, respectively) compared to that in the parental TK24. *S. lividans* ΔYA10 and ΔYA11, containing one and two additional *attB* sites, respectively, showed a further increase in the accumulation of deoxycinnamycin. Among all tested strains, the highest productivity was observed in *S. lividans* ΔYA10, with a 4.5-fold (SG) and 3.5-fold (DNPM) increase compared to that in the wild type. In the case of *S. albus* Del14, accumulation of deoxycinnamycin was slightly lower than or on par with that in *S. lividans* ΔYA9, while *S. coelicolor* M1154 produced less than the other tested strains.

In the case of the aromatic polyketide griseorhodin, surprisingly, no production was observed by *S. lividans* TK24 and *S. lividans* ΔYA11 when cultivated in SG medium, which was originally developed for polyketide production (Fig. 4c). Two other *S. lividans* strains, ΔYA9 and ΔYA10, were able to produce this antibiotic in relatively low amounts. *S. albus* Del14 showed the highest production level of griseorhodin in SG medium. A different behaviour was observed when strains were cultivated in DNPM medium. While *S. lividans* TK24 still lacked the production of griseorhodin, strain ΔYA9 that is deficient in a large part of its own secondary metabolism was able to produce griseorhodin. The production level was further increased with the use of strains carrying additional *attB* sites (twofold in ΔYA10 and almost fourfold in ΔYA11 compared to that with *S. lividans* ΔYA9). However, similar to tunicamycin, the highest accumulation of griseorhodin was observed in *S. albus* Del14 in both media. For *S. coelicolor,* no ex-conjugants were obtained with the griseorhodin gene cluster construct after 20 attempts.

### Expression of the BAC library of *S. albus* subsp. *chlorinus* NRRL B-24108

**Fig. 5:**
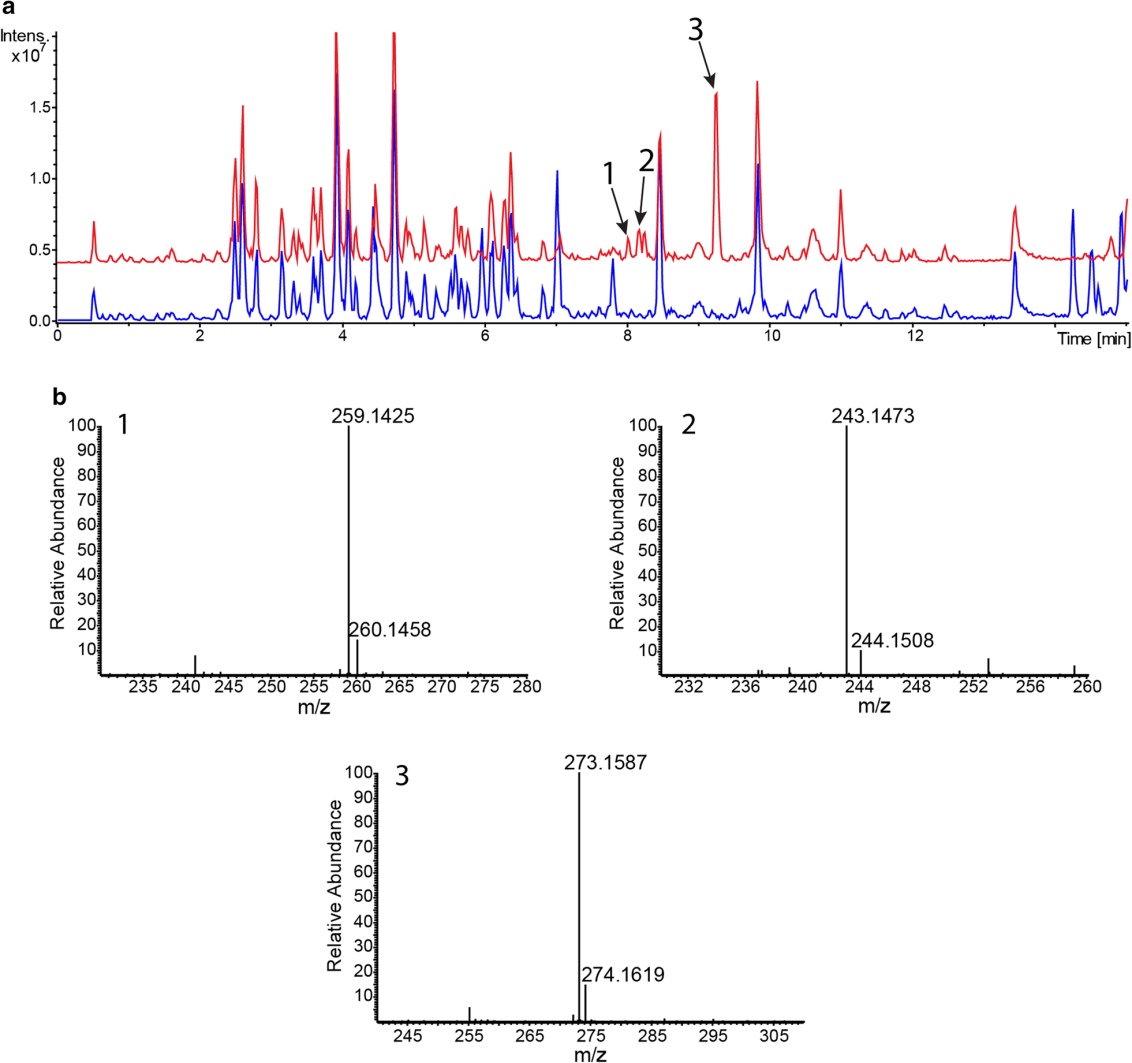
LC-MS analysis of *S. lividans* ΔYA9 with and without BAC2I4. Base peak chromatogram of ethyl acetate extracts of *S. lividans* ΔYA9 (blue) and *S. lividans* ΔYA9/BAC2I4 (red) grown in DNPM medium for 6 days. The new PBDs are marked as **1** (RT 8.0 min, *m/z* 259.14 [*M*+H]^+^), **2** (8.2 min, *m/z* 243.14 [*M*+H]^+^) and **3** (9.3 min, *m/z* 273.15 [*M*+H]^+^). Samples were separated with the 20 min gradient protocol

The big advantage of engineered *Streptomyces* host strains is that a simplified metabolic background allows for fast identification of compounds produced by expressing unknown biosynthetic gene cluster(s). To test the feasibility of the developed strains, we used *S. albus* Del14 and *S. lividans* ΔYA9 to screen the BAC library of *S. albus* subsp. *chlorinus* NRRL B-24108 [41] for the production of new secondary metabolites. For this purpose, 17 clones carrying secondary metabolite gene clusters predicted by antiSMASH were systematically expressed in *S. lividans* ΔYA9 and *S. albus* Del14, and extracts were screened for new compounds by LC–MS. As a result, six clones were successfully expressed either in *S. lividans* (two clones) or *S. albus* Del14 (five clones), resulting in the appearance of new peaks on the LC–MS chromatogram of corresponding extracts (Table 2). One cluster encoding a type II PKS with unknown products was successfully expressed in both strains. The other five were active either in the *S. lividans* or *S. albus* host. Among the compounds produced by *S. albus* Del14 was the promising antimycobacterial compound nybomycin [42]. On the other hand, BAC clone 2I4 was expressed only in *S. lividans* ΔYA9, leading to the accumulation of three new compounds with RTs of 8.0 min (**1**), 8.2 min (**2**), 9.3 min (**3**) and *m/z* values of 259.1425 [*M*+H]^+^, 243.1473 [*M*+H]^+^ and 273.1587 [*M*+H]^+^, respectively (Fig. 5). The BAC 2I4 clone harbours an uncharacterized gene cluster with a predicted NRPS core enzyme showing a high degree of homology to several members of the pyrrolobenzodiazepine (PBD) biosynthesis NRPSs, such as those involved in the biosynthesis of anthramycin and porothramycin [43, 44] (Additional files 4, 5 and 6: Figs. S3, S4 and Table S2, respectively). Based on the exact mass, the closest DNP hits for the identified compounds are usabamycin A (calculated *m/z* 273.15583 [*M*+H]^+^), B (calculated *m/z* 259.14018 [*M*+H]^+^) and C (calculated *m/z* 243.14527 [*M *+ H]^+^) (Fig. 5, Additional file 5: Fig. S4). However, the MS/MS patterns of the produced compounds significantly differed from those predicted for usabamycins since all three compounds lose water during fragmentation (Additional file 7: Fig. S5). Despite the fact that the exact nature of these metabolites still has to be established by more precise structural analysis, we can clearly say that they are new representatives of the PBD family of natural products.

**Table 2:** Expression of *S. albus* subsp. *chlorinus* NRRL B-24108 BAC library clones in engineered host strains

BAC	Type of cluster	*S. lividans* ΔYA9	*S. albus* Del14
4M20	Type I PKS	−	−
2O18	NRPS/siderophore	−	+
6O24	NRPS/siderophore	−	−
5A14	Siderophore	−	−
2P5	Type II PKS	+	+
1P15	Lantipeptide	−	−
4E8	NRPS	−	+
3D8	Siderophore	−	−
6E10	Type I PKS	−	−
3N1	Lantipeptide	−	−
1K1	NRPS	−	−
5K5	Lantipeptide	−	−
5F24	Phenazine	−	+
5H22	NRPS	−	−
2I4	NRPS	+	−

**Fig. 6:**
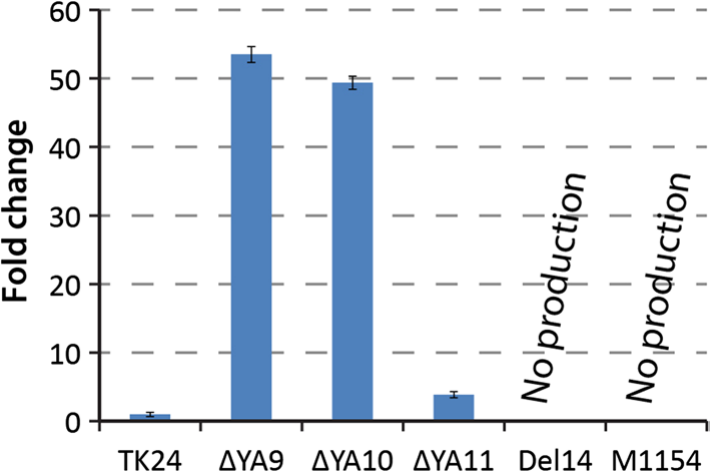
Production levels of PDB-like compound 3 in different hosts. The recombinant colonies were grown for 6 days in DNPM. The production by *S. lividans* TK24 was taken as 100%

To investigate the influence of copy number on the production of identified PBDs, we introduced 2I4 BAC clones into *S. lividans* TK24, ΔYA9, ΔYA10 and ΔYA11. Strains were grown in DNPM, and metabolites were extracted and analysed with LC–MS. As a result, the two engineered *S. lividans* strains ΔYA9 and ΔYA10 showed 40-fold and 60-fold increases in accumulation of PBD compounds, respectively (Fig. 6). Furthermore, the parental strain produced trace amounts of these compounds. The strain with three *attB* sites, ΔYA11, demonstrated a twofold elevation in production level when compared to that of the parental TK24 strain that might be caused by frequent loss of the gene cluster due to recombination events.

## Discussion

Despite the accessibility of a large number of actinobacterial strains, only several of them, *S. coelicolor*, *S. lividans* and *S. albus,* are well studied in terms of general physiology, biochemistry and genetics and thus are used as heterologous hosts for antibiotic production. In rare cases, some industrial strains or their derivatives are also used as expression hosts. In all cases, industrial and general strains have advantages and disadvantages, including poor genetic tractability (e.g., *S. coelicolor*), a high level of production of endogenous metabolites (e.g., industrial hosts), limited precursor supply (e.g., general hosts), cross-regulation and biochemical interactions between endogenous and heterologous gene clusters. [9]. As a solution to some of these problems, several attempts to modify *Streptomyces* strains for increased production of exogenous secondary metabolites have been reported [11, 12, 15]. In all cases, endogenous secondary metabolism was targeted to minimize the genome of the host to improve the fitness and growth rate as well as redirect precursors and regulatory factors to the heterologous biosynthetic pathway. As such, engineered strains of *S. albus*, *S. coelicolor*, *S. avermitilis*, and *S. chattanoogensis* were generated [45]. The engineering of such “clean” metabolic background strains is of high importance first and foremost since they allow the shift from chemical to bioinformatics dereplication of bacterial natural products. The dereplication step is required in current natural product discovery projects due to the frequent re-discovery of the same compounds. It is used to eliminate tedious and labourious processes of compound isolation and structure elucidation by applying high-resolution MS techniques. However, chemical dereplication often excludes true hits. With advances in genome sequencing, the identification of a gene cluster can be used as another criterion in the dereplication process [46]. However, regarding heterologous expression of genomic or metagenomic libraries, the dereplication can be entirely shifted from chemical to bioinformatics by analysing the sequence and architecture of gene clusters to preselect the ones with unique features. Expressing such pre-selected gene clusters in the host with a well-defined or preferably “clean” secondary metabolite background will provide increased chances for discovery of new natural products.

To expand the range of such host strains, herein, we report the development of new *S. lividans*-based chassis strains for heterologous expression of secondary metabolites gene clusters. On top of the balanced and amenable *S. lividans* TK24 strain, we worked our way towards a simplified metabolic background by deleting nine endogenous gene clusters while keeping track on the strains’ fitness. We further engineered our streamlined strain to include two additional sites for integration of foreign DNA in place of two endogenous secondary metabolites gene clusters. The engineered strain efficiency in the production of secondary metabolites was validated by expressing four heterologous gene clusters to produce secondary metabolites of different natures.

The general cloning strains that, in recent decades, were (and still are) a preferred choice for heterologous gene cluster expression are *S. lividans* TK24, *S. coelicolor* M415, and *S. albus* J1074. Later, two became a basis for the generation of engineered chassis strains by inactivating a large portion of endogenous secondary metabolism. Additionally, there have been several attempts to generate such hosts based on *S. lividans* TK24 by deleting *act* and *red* gene clusters [21, 22]; however, no systematic engineering of the strain to create a “clean” background host has been reported to date. This study is important first and foremost due to the fact that undecylprodigiosin and especially actinorhodin are produced by *S. lividans* only at certain and very limited number of conditions when other secondary metabolites obviously have more impact on heterologous natural product biosynthesis [13]. With its fast growth and ease of handling in general, *S. lividans* is a preferable host strain for the heterologous production of proteins and secondary metabolites.

To fill the gap in the range of engineered hosts, we have chosen to delete eleven gene clusters from the chromosome of *S. lividans* TK24. Typically, two different approaches are considered in chassis strain construction. The first one involves a deletion of either an empirically chosen large region of chromosome that also includes several gene clusters for secondary metabolites and avoids the core genome region. This method is often accompanied by bioinformatic analysis of the essentiality of the genes in selected regions to avoid fitness costs [11, 45]. In this way, *S. avermitilis* and *S. chattanoogensis* chassis strains were generated. Different approaches were used to create the *S. coelicolor* M1154 and *S. albus* Del14 strains [12, 15]. In this case, a precise selection and sequential deletion of a set of gene clusters for endogenous secondary metabolite biosynthesis was performed. This approach avoids any problems with the loss of genes important for growth, regulation and morphological development and produces the chassis strain with a clean metabolic profile.

To generate *S. lividans* chassis strains, the gene clusters were selected for deletion based on high transcriptional level and positive detection of associated compounds in production media. As a result, all PKS- and NRPS-encoding clusters were removed from the chromosome of the strain except for the type II PKS *whiE* gene cluster (number 12), which is responsible for the spores’ pigment biosynthesis [47]. The *whiE* cluster is ectopically expressed in aerial mycelium immediately before sporulation, and since *S. lividans* does not sporulate in liquid culture, it is not in competition for CoA substrates. The ectoine and deferoxamine biosynthesis gene clusters were also kept in the genome of engineered strains because of the essential role of these compounds in physiological responses to growth under osmotic stress and low-iron conditions, respectively [26, 48]. Deletion of the deferoxamine biosynthesis gene cluster had a deleterious effect in *S. albus*, showing its essentiality even under normal growth conditions [32]. A similar effect was observed when we attempted to delete a gene cluster annotated as involved in the biosynthesis of terpene (number 3). Despite being able to obtain double crossover clones, the clones were found to grow poorly after re-plating on fresh medium. This gene cluster showed 100% similarity to the hopene biosynthesis genes in *S. coelicolor* [49]. Hopanoids are produced by *S. coelicolor* during the transition from substrate to aerial mycelium growth on solid medium but are not accumulated in liquid culture [50]. These compounds are components of the cytoplasmic membrane, and their function is to reduce the stress in aerial hyphae by decreasing the water permeability across the cell membrane. Hopanoids have been reported to be not essential for the growth of *S. scabies* [51], although we have observed their strong influence on the growth of *S. lividans* lacking the respective gene cluster. Thus, cluster 3 remained within the genomes of engineered strains. Out of the remaining 5 terpene gene clusters, we deleted cluster 13 coding for albaflavenone biosynthesis [52]. Two gene clusters (number 23 and 25, coding for biosynthesis of isorenieratene and isoprenoid, respectively) are not covered by the BAC clones in the used *S. lividans* genomic library [30]. Two remaining terpene clusters encode for geosmin (number 8) and uncharacterized terpene (number 1, absent in *S. coelicolor* genome) biosynthetic enzymes and were left in the engineered strains. One of the major contaminants in the extract of *S. lividans* TK 24 is melanin, which gives dark colour to the media and interferes with downstream processing of the produced compound of interest, especially after long cultivation. This fact led us to include the melanin gene cluster (number 17) as one of the major candidates for deletion despite the low level of its transcription under tested conditions.

For actinobacteria genome engineering, mostly the Cre/*loxP* recombination system has been used [53–56]. However, after the recombination event, it leaves an active scar that can be recognized by a recombinase, thus significantly complicating the next engineering step due to large chromosomal rearrangements. To generate *S. lividans* strains, the IMES system was used [32]. This system allows recycling of the resistance marker in the same genomic background without any interference with the remaining scar in the chromosome. As a result, we constructed a set of strains, *S. lividans* ΔYA 9, *S. lividans* ΔYA10 and *S. lividans* ΔYA11, carrying deletions of 9, 10, and 11 endogenous gene clusters, respectively, with the last two strains harbouring additional *attB* sites for heterologous cluster integration. In addition to the clean metabolic background, these strains had slightly better growth characteristics in liquid production medium, possibly due to the smaller chromosome size and lack of materials and energy draining into secondary metabolism.

To assess the usefulness of the engineered *S. lividans* strains as a heterologous host in comparison with the well-established chassis strains, such as *S. coelicolor* M1154 and *S. albus* Del14, we introduced several characterized gene clusters into these strains. As a general observation, the conjugation frequency in all tested strains was different. In the case of *S. albus* Del14, *S. lividans* TK24 and *S. lividans* ΔYA9, the conjugation efficiency was comparable. However, the introduction of additional *attB* sites impaired gene transfer in the case of *S. lividans* ΔYA10 and *S. lividans* ΔYA11. Similar behaviour was observed in a previous study by our group, when the *S. albus* SAM2 strain with only one *attB* site in the chromosome demonstrated the highest conjugation efficiency when compared to that of other *S. albus* strains with multiple integration sites [57]. In the case of *S. albus*, this phenomenon was postulated to be not caused by recombination between different *attB* sites within the same genome but was rather related to the efficiency of the integrase enzyme to simultaneously integrate several copies of the vector DNA. However, a scenario in which the recombination between the integrated sequences occurs cannot be excluded. In the original study, the pSET152 vector was used, which is only 5.5 kb in size. The gene clusters provide a much larger template for recombination that might result in deletion of a part of the chromosome, leading to lethality of some portion of transconjugants.

Although the engineered *S. lividans* strains performed better than the parental TK24, they also demonstrated a larger degree of deviation in production level between clones. The degree of instability increased with the increase in the number of *attB* sites in the strains’ chromosomes. This result suggests that these two factors are related, and the deviation between different clones could be caused by recombination events, eliminating part of the integrated gene clusters. Another reason for the observed deviation might be the difference in efficiency of integration into *attB* and pseudo *attB* sites, resulting in different numbers of gene clusters being integrated into chromosomes of individual clones. It is a well-established fact that the integration of φC31-based vectors in *S. lividans* TK24 into different *attB* sites occurs with different efficiencies [58]. Independent of the reasons, the *S. lividans* ΔYA10 and especially the ΔYA11 transconjugant population obviously had a high degree of heterogeneity, resulting in dramatic variation in the production level from clone to clone and in long-term cultivation. Thus, care should be taken when selecting the proper strain and individual clone(s) for scaled up production of a desired compound in the case of the ΔYA10 and ΔYA11 strains.

The engineered *S. lividans* strains were found to perform better with the gene clusters encoding biosynthesis of amino acid-derived compounds, such as deoxycinnamycin and the identified PBD family natural product. The latter metabolite was not produced by *S. albus* Del14 when the respective construct was introduced. It is also well known that *S. lividans* is a long-term preferred host for ribosomally synthesized and post-translationally modified peptide (RiPP) gene cluster expression [59, 60]. At the same time, the polyketide griseorhodin was not produced by *S. lividans* TK24 at all, and *S. albus* Del14 was superior to all other strains tested in this work. Similarly, the pamamycin gene cluster was successfully expressed solely in *S. albus* but not in any other strain tested [61]. Deletion of endogenous secondary metabolites gene clusters in *S. lividans* resulted in activation of griseorhodin biosynthesis in the *S. lividans* ΔYA9, ΔYA10 and ΔYA11 strains, with an obvious copy-number effect observed. The reasons for this result are not very clear; perhaps the sink of precursors into internal polyketide pathways for Act, Red, coelimycin, and herboxidiene resulted in high competition and thus lack of griseorhodin production. Additionally, the interplay between regulatory systems controlling the expression of secondary metabolism in *S. lividans* cannot be excluded. In the case of *S. albus,* the deletion of endogenous secondary metabolites gene clusters resulted in elevated transcription levels of heterologous biosynthetic genes, clearly indicating the possibility of such interactions [15]. Furthermore, we recently demonstrated that the *S. albus* butenolide regulatory system influences the production of heterologous secondary metabolites [62]. It is difficult to say if this situation is true also for *S. lividans*, but at least in the case of griseorhodin, the phenotype is very prominent, pointing to the possibility of such regulatory interplay. A similar phenomenon was observed in the case of deoxycinnamycin and PBD-like compound production, when the accumulation of both compounds was significantly elevated in the ΔYA9 strain when compared with that in TK24. In fact, we would most likely not be able to detect the PBD compounds if the library was screened in the parental strain.

The clean metabolic background of engineered strains provides another benefit in their utilization as hosts for screening genomic and metagenomic libraries for new secondary metabolites. The absence of endogenously produced compounds significantly improves the detection and identification of products. Moreover, it is quite obvious that the success of such projects strongly depends on the efficiency of the utilized chassis strain(s). As such, griseorhodin was produced in *S. albus* Del14 but not in *S. lividans* TK24. Conversely, the expression of a gene cluster for the potent ribosome inhibitor bottromycin was successful in *S. lividans* but not *S. albus* [63]. In fact, there are many examples when a gene cluster was expressed in one but not another host strain. Unfortunately, there is no universal chassis strain for such experiments. Thus, the success rate of genomic or metagenomics library screening for new secondary metabolites will strongly benefit from the use of more than one expression host. The engineered *S. lividans* strains complement the panel of metabolically clean heterologous gene cluster expression chassis strains.

## Conclusion

In conclusion, the developed strains represent a step forward towards a better panel of organisms for bioprospecting and genome mining of novel natural products. In fact, it is becoming obvious that different chassis stains have a preference for certain types of natural products. For example, *S. albus* is a successful host for the expression of polyketide secondary metabolites, while *S. lividans* performs better with gene clusters coding for the production of amino acid-based natural products, such as RiPPs and nonribosomal peptides. However, despite the obvious progress in this direction, the current panel of expression hosts is obviously far from being able to satisfy the needs of screening programmes and still would have to be expanded initially with non-streptomycete actinobacteria strains to achieve a high success rate in new natural product identification and isolation.

## Methods

### Strains, plasmids and growth conditions

All bacterial strains, plasmids and BACs used in this study are listed in Additional file 8: Table S3. *E. coli* strains were grown in LB (lysogeny broth) medium [64]. *Streptomyces* strains were grown on mannitol soy flour agar (MS agar) [65] for spores formation and in liquid tryptic soy broth (TSB) medium for pre-culture (Sigma-Aldrich, USA). Modified SG (glucose 20 g, yeast extract 5 g, soytone 10 g, calcium carbonate 2 g, dH_2_O 1000 ml) and DNPM [66] (dextrin 40 g, soytone 7.5 g, fresh yeasts 5 g, MOPS 21 g, dH_2_O 1000 ml, pH 6.8) were used for secondary metabolites production. The following antibiotics were used when necessary: apramycin (50 µg/ml), kanamycin (30 µg/ml), hygromycin (50 µg/ml), thiostrepton (50 µg/ml) fosfomycin (100 µg/ml) and chloramphenicol (50 µg/ml) (Carl Roth, Germany, and Sigma-Aldrich, USA).

### Isolation and manipulation of DNA

DNA isolation and manipulation, *E. coli* transformation and *E. coli*/*Streptomyces* intergeneric conjugation were performed according to standard protocols [64, 65, 67]. Dream Taq polymerase (Thermo Fisher Scientific, USA) was used to amplify DNA fragments for cloning, for PCR verification of constructs and strains. DNA fragments were purified from agarose gels using the *QIAquick Gel Extraction* Kit (Qiagen, Germany). Restriction enzymes and ligase were used accordingly to manufacturer recommendations (New England Biolabs, USA). All primers used in this study are listed in Additional file 9: Table S4 (Eurofins Genomics, Germany).

### Construction of *S. lividans* TK24 mutants

To delete the desired gene clusters BAC clones (Additional file 8: Table S3) from ordered *S. lividans* genomic library were selected [30] and modified using Red/ET recombination approach combined with IMES antibiotic resistance cassettes [31, 32]. The IMES cassette containing apramycin resistance marker and *oriT* was excised from a carrier plasmid (patt-saac-*oriT*) with *Pvu*II and amplified using primers listed in (Additional file 9: Table S4). Red/ET was performed as previously described [68]. Deletions were confirmed by PCR using check primers listed in Additional file 9: Table S4. The recombinant BACs were introduced stepwise into the *S. lividans* strains. The double-crossover mutants were screened on MS medium supplemented with apramycin and 50 μg/ml of X-Gluc (X-CLUC Direct, USA). After each deletion the resistance marker was removed from the chromosome of the generated strains by expression of φC31 actinophage integrase [32], and the resulting strains genotype was confirmed by PCR and PCR product sequencing.

### Introduction of additional *attB*-sites into the chromosome of *S. lividans* ΔYA9

To introduce *attB* sites into *S. lividans* strains the synthetic cassette consisting of hygromycin resistance marker flanked by *Mss*I restriction sites and *attB* sequence was used [15]. This cassette was PCR amplified using primers for Red/ET Additional file 9: Table S4. Large fragments of clusters 21 and 5 were replaced with the above mentioned cassettes in BACs 1468 and 1092, respectively using Red/ET recombination. Hygromycin resistance gene was removed by digesting with *Mss*I restriction enzyme followed by self-ligation. The resulting BACs were named 1468::attB and 1092::attB, respectively. Recombinant BACs were further modified by substituting the chloramphenicol gene (*cat*) with cassette containing apramycin resistance gene (*aac(3)IV*) for selection in *Streptomyces* and *oriT* sequence to conjugation transfer of construct from *E. coli* to *Streptomyces*. The final BACs (1468::attBamoriT and 1092::attBamoriT, respectively) were introduced sequentially into *S. lividans* ΔYA9 strain by conjugation. The secondary cross-over colonies were selected by lack of β-glucuronidase activity and apramycin sensitivity phenotype caused by loss of the vector backbone. The deletion of the gene clusters and introducing additional *attB* sites were confirmed by PCR using primers listed in Additional file 9: Table S4. Additionally, the PCR products were sequenced to confirm presence and correct sequence of *attB* sites.

### Growth and sporulation study

For biomass measurement the wild type *S. lividans* TK24 and the obtained strains, *S. lividans* ΔYA9 and *S. lividans* ΔYA11 were grown in 20 ml of TSB for 2 days. 12 mg of biomass of each pre-culture was used to inoculate 50 ml of DNPM production medium. 1 ml of each sample was taken every 12 h for 3 days. The biomass was harvested by centrifugation for 5 min at 14,000 rpm. The supernatant was discarded, and the weight of dry biomass was measured.

### Heterologous expression of secondary metabolite gene clusters

Constructs carrying gene clusters for biosynthesis of the nucleoside antibiotic tunicamycin, the aromatic polyketide griseorhodin, the lantipeptide deoxycinnamycin (Additional file 8: Table S3) were introduced into *S. lividans* TK24, *S. lividans* ΔYA9, *S. lividans* ΔYA10, *S. lividans* ΔYA11, *S. albus* Del14 and *S. coelicolor* M1154 by mean of intergeneric conjugation [65].

The genomic library of *S. albus* subsp. *chlorinus* NRRL B-24108 was constructed on pSMART vector by Intact Genomics, USA. First, 17 clones were introduced into *S. lividans* ΔYA9 and *S. albus* Del14. Resulting strains were cultivated and the metabolites were extracted and analysed as described below. Out of 17 clones the BAC2I4 was chosen for further study and was introduced into above-mentioned tested hosts by conjugation.

### Cultivation conditions and extraction of secondary metabolites

Three independent clones of each recombinant strain were grown in 30 ml of TSB for 2 days (except *S. albus* Del14 were grown for 24 h) and 100 mg of biomass of the pre-culture was used to inoculate 50 ml of production media. Strains were cultivated for 6 days at 28 °C and metabolites were extracted as following: (1) the deoxycinnamycin was extracted with butanol; (2) tunicamycins were extracted with butanol and acetic acid; (3) the PBD-like compounds were extracted with ethyl acetate; (4) griseorhodin was extracted with ethyl acetate and acetic acid. Extracts were evaporated and dissolved in 300 µl of DMSO/MeOH (1:1). 3 µl were analyzed by LC-MS as described below. Metabolites accumulation was averaged and the production by *S. lividans* TK24 was taken as 100% in each particular experiment (except for griseorhodin the *S. lividans* ΔYA9 was taken as 100%).

### Analysis of secondary metabolites production

3µL of each sample was separated using a Dionex Ultimate 3000 UPLC (Thermo Fisher Scientific, USA) and a 10-cm ACQUITY UPLC^®^ BEH C18 column, 1.7 μm (Waters, USA) and a linear gradient of acetonitrile against 0.1% formic acid solution in water from 5% to 95% in 10 or 18 min at a flow rate of 0.6 ml/min. Samples were analyzed using an amaZon speed mass spectrometer (Bruker Daltonics, Germany) using ESI source. Mass spectra were acquired in centroid mode ranging from 200 to 2000 *m/z* at a 2 Hz scan rate. Data was collected and analyzed with the Bruker Compass Data Analysis software, version 4.2 (Bruker, Billerica, USA). For determination of accurate mass Thermo LTQ Orbitrap XL coupled to UPLC Thermo Dionex Ultimate 3000 RS was used. Data were acquired with Xcalibur 2.2 software (Thermo Scientific). The separation conditions were identical to those used for quantification study. For fragmentation pattern the mass spectra were ranging from 50 to 2000 *m/z*.

### Purification of compounds **4a–d**

*Streptomyces lividans* ΔYA11 was grown at 30 °C for 3 days in 6 × 500-ml flasks containing 50 ml of TSB, and pre-culture was used to inoculate 100× 500-ml flasks containing 50 ml of SG media. Cultures were incubated at 30 °C for 6 days. Metabolites were extracted as described above. The extracts from biomass and the supernatant were combined and fractionated by size-exclusion chromatography on an LH 20 Sephadex column (Sigma-Aldrich, USA) using methanol as the solvent. The fractions were collected every 15 min, evaporated and dissolved in 0.5 ml of MeOH. The obtained fractions with mixture of compounds 4 were further purified by Agilent 1260 Series from Agilent Technologies (semipreparative). Semipreparative HPLC was performed using a Jubiter LC-Column (250 × 10 mm, 4 μm; Phenomenex) with a multistep gradient from 5–80% B (B: acetonitrile with 0.1% formic acid;A: water with 0.1% formic acid) over 5 min and stay constants at 80% B over 10 min, afterwards from 80–95% B over 5 min at a flow rate of 4 ml/min and 45 °C.

### Nuclear magnetic resonance (NMR) spectroscopy and structure elucidation of **4a**–**d**

^1^H-NMR and 2D HHCOSY, HSQC and HMBC were recorded on a Bruker Avance III 700 spectrometer (Bruker, BioSpin GmbH, Rheinstetten, Germany) at 298 K equipped with a 5 mm TCI cryo probe using deuterated methanol (Deutero, Kastellaun, Germany) as solvent. The chemical shifts were reported in parts per million (ppm) relative to the solvent peaks (δ_H_ 3.30 and δ_C_ 49.00, respectively). All spectra were performed using standard pulse programs from the Bruker pulse program library.

The mass spectra taken from the BPC chromatogram of *S. lividans* TK24 extract (Additional file 2: Fig. S1) showed a set of at least four members of a homologous series with [M+H]^+)^ = 597.58, 611.58, 625,62, 639,64 amu (**4a**–**d**). Purification of the extract led to enriched fractions of **4b** and **4c**, which could be subjected to NMR studies. Both ^1^H NMR [Additional file 10: Fig S6, S(6-1), S(6-2) and S(6-3)] were very close to each other and differed only in the integration of a few resonances. Intensive research and careful comparison of the data with those from literature [69] revealed a 1-monoglyceride structure (δ_H_ 4.14 dd, J = 11.5 and 4.5 Hz, 4.05 dd, J = 11.5 and 6.5 Hz: ROC**H**_2_-CHOH-CH_2_OH; δ_H_ 3.81 m: ROCH_2_-CHO**H**-CH_2_OH; δ_H_ 3.55 dd and 3.53, both J = 11.5 and 5.5 Hz: ROCH_2_-CHOH-C**H**_**2**_OH). In contrast to ordinary fats the fatty acids in **4b** and **4c** showed resonances for a hydroxy group. Their chemical shift (δ_H_ 5.21) proved an esterification at this position. The acid substructure of this ester remained unidentified, mainly due to signal overlapping. The small amount of substance did not allow a needful chemical degradation, purification and further NMR investigations.

## Supplementary information

**Additional file 1: Table S1.** Secondary metabolites gene clusters in *S. lividans* TK24 and their transcriptional level Average in minimal medium in mini-bioreactor [1].

**Additional file 2: Fig. S1.** BPC chromatogram extract of *S. lividans* TK24.

**Additional file 3: Fig. S2.** Proposed structures for 4a–d.

**Additional file 4: Fig. S3.** Comparison of *S. albus* subsp. *chlorinus* NRPS gene cluster with described PBD biosynthetic gene clusters.

**Additional file 5: Fig. S4.** Structures of several representatives of pyrrolobenzodiazepines (PBDs) family.

**Additional file 6: Table S2.** Homologous genes in described PBD biosynthetic gene clusters.

**Additional file 7: Fig. S5.** Fragmentation pattern of compounds identified in the extract of *S. lividans* ΔYA9 with BAC2I4.

**Additional file 8: Table S3.** Strains, Plasmids and BACs used in this study.

**Additional file 9: Table S4.** Primers used in this study.

**Additional file 10: Fig. S6.** ^1^H NMR overview spectrum.

## Data Availability

(1) The datasets and materials used and/or analyzed during the current study are available from the corresponding author on reasonable request. (2) All data generated or analyzed during this study are included in this published article.

## Abbreviations

IMES: iterative marker excision system
NRPS: nonribosomal peptide synthetases
PBD: pyrrolobenzodiazepines
PKS: polyketide synthetases
RPKM: reads per kilo base per million mapped reads
LC–MS: liquid chromatography mass spectrometry
BAC: bacterial artificial chromosome
DNP: Dictionary of Natural Products
RiPPs: ribosomally synthesized and post-translationally modified peptides
