# Supplementary material for: Engineering of Streptomyces lividans for heterologous expression of secondary metabolite gene clusters
Source: Microb Cell Fact. 2020 Jan 9;19:5. doi: 10.1186/s12934-020-1277-8 (PMC6950998; doi:10.1186/s12934-020-1277-8)
Supplement: Supplementary file 1 — Additional file 1: Table S1. Secondary metabolites gene clusters in S. lividans TK24 and their transcriptional level Average in minimal medium in mini-bioreactor [1]. [file 12934_2020_1277_MOESM1_ESM.docx]

**Additional file 1**

**Engineering of *Streptomyces lividans* for heterologous expression of secondary metabolite gene clusters**

Yousra Ahmed^1^, Yuriy Rebets^1^, Marta Rodríguez Estévez^1^, Josef Zapp^2^, Maksym Myronovskyi^1^, Andriy Luzhetskyy^1, 3,^*****

^1^Pharmazeutische Biotechnologie, Universität des Saarlandes, Saarbrücken, Germany

^2^Pharmazeutische Biologie, Universität des Saarlandes, Saarbrücken, Germany

^3^Helmholtz-Institut für Pharmazeutische Forschung Saarland, Saarbrücken, Germany

***Correspondence:** [**a.luzhetskyy@mx.uni-saarland.de**](mailto:a.luzhetskyy@mx.uni-saarland.de)**.**

A full list of author information is available at the end of the article.

**Table. S1.** Secondary metabolites gene clusters in *S. lividans* TK24 and their transcriptional level

Average in in minimal medium in mini-bioreactor [[1](#_ENREF_1)].

| Cluster | Name | Coordinates, bp | ORFs | RPKM |
| --- | --- | --- | --- | --- |
| Cluster 1 | terpene | 16255 - 39174 | SLIV_00085 - SLIV_00185 | 17.5 |
| Cluster 2 | coelibactin | 145838 - 218926 | SLIV_00745 - SLIV_01060 | 198.4 |
| Cluster 3 | terpene | 1013598 - 1039716 | SLIV_04650 - SLIV_04765 | 205.6 |
| Cluster 4 | lantipeptide | 1116997 - 1135734 | SLIV_05075 - SLIV_05165 | 157.8 |
| cluster 5 | NRPS | 1380581 - 1429153 | SLIV_06175 - SLIV_06340 | 62.1 |
| Cluster 6 | coelimycin | 1504477 - 1583755 | SLIV_06690 - SLIV_06900 | 33.4 |
| Cluster 7 | siderophore | 1601312 - 1613393 | SLIV_06985 - SLIV_07035 | 36.1 |
| Cluster 8 | terpene | 1777266 - 1799442 | SLIV_07735 - SLIV_07820 | 109.3 |
| Cluster 9 | bacteriocin | 1811603 - 1821470 | SLIV_07885 - SLIV_07930 | 294.4 |
| Cluster 10 | undecylprodigiosin | 2068946 - 2116414 | SLIV_09055 - SLIV_09240 | 91.1 |
| Cluster 11 | siderophore | 2198827 - 2209792 | SLIV_09600 - SLIV_09630 | 918.1 |
| Cluster 12 | type II PKS | 2688586 - 2730052 | SLIV_11695 - SLIV_11900 | 325.5 |
| Cluster 13 | terpene | 2804344 - 2825931 | SLIV_12210 - SLIV_12310 | 803.1 |
| Cluster 14 | actinorhodin | 2944091 - 2985628 | SLIV_12835 - SLIV_13045 | 351.4 |
| Cluster 15 | CDA | 4737997 - 4818586 | SLIV_21425 - SLIV_21620 | 53.9 |
| Cluster 16 | siderophore | 5289644 - 5301384 | SLIV_23725 - SLIV_23770 | 844.3 |
| Cluster 17 | melanin | 5385376 - 5394552 | SLIV_24140 - SLIV_24195 | 94.1 |
| Cluster 18 | ectoine | 6336179 - 6347102 | SLIV_28360 - SLIV_28410 | 360.3 |
| Cluster 19 | germicidin | 7040159 - 7082308 | SLIV_31665 - SLIV_31865 | 226.3 |
| Cluster 20 | bacteriocin | 7572312 - 7580633 | SLIV_34155 - SLIV_34180 | 35.9 |
| Cluster 21 | coelichelin | 7827171 - 7878351 | SLIV_35400 - SLIV_35580 | 707.8 |
| Cluster 22 | lantipeptide | 8101385 - 8137004 | SLIV_36525 - SLIV_36675 | 323.1 |
| Cluster 23 | terpene | 8180179 - 8205399 | SLIV_36890 - SLIV_37000 | 398 |
| Cluster 24 | eicosapentaenoic acid | 8242078 - 8295559 | SLIV_37190 - SLIV_37380 | 38.1 |
| Cluster 25 | terpene | 8308420 - 8328145 | SLIV_37430 - SLIV_37535 | 6.9 |
| RNA polymerase β- subunit | | 3408742 - 3404843 | SLIV_25060 | 958.5 |
| *S. lividans* TK24 genome average RPKM | | | | 426.4 |

**References**

1. Busche T, Tsolis KC, Koepff J, Rebets Y, Ruckert C, Hamed MB, Bleidt A, Wiechert W, Lopatniuk M, Yousra A *et al*: **Multi-Omics and Targeted Approaches to Determine the Role of Cellular Proteases in *Streptomyces* Protein Secretion**. *Front Microbiol* 2018, **9**:1174.

2. Ruckert C, Albersmeier A, Busche T, Jaenicke S, Winkler A, Friethjonsson OH, Hreggviethsson GO, Lambert C, Badcock D, Bernaerts K *et al*: **Complete genome sequence of *Streptomyces lividans* TK24**. *J Biotechnol* 2015, **199**:21-22.

3. Myronovskyi M, Rosenkranzer B, Nadmid S, Pujic P, Normand P, Luzhetskyy A: **Generation of a cluster-free *Streptomyces albus* chassis strains for improved heterologous expression of secondary metabolite clusters**. *Metab Eng* 2018.

4. Gomez-Escribano JP, Bibb MJ: **Engineering *Streptomyces coelicolor* for heterologous expression of secondary metabolite gene clusters**. *Microb Biotechnol* 2011, **4**(2):207-215.

5. Flett F, Mersinias V, Smith CP: **High efficiency intergeneric conjugal transfer of plasmid DNA from Escherichia coli to methyl DNA-restricting streptomycetes**. *Fems Microbiol Lett* 1997, **155**(2):223-229.

6. Blodgett JA, Thomas PM, Li G, Velasquez JE, van der Donk WA, Kelleher NL, Metcalf WW: **Unusual transformations in the biosynthesis of the antibiotic phosphinothricin tripeptide**. *Nat Chem Biol* 2007, **3**(8):480-485.

7. Fu J, Teucher M, Anastassiadis K, Skarnes W, Stewart AF: **A Recombineering Pipeline to Make Conditional Targeting Constructs**. *Method Enzymol* 2010, **477**:125-144.

8. Myronovskyi M, Rosenkranzer B, Luzhetskyy A: **Iterative marker excision system**. *Appl Microbiol Biotechnol* 2014, **98**(10):4557-4570.

9. Rebets Y, Tsolis KC, Guðmundsdóttir EE, Koepff J, Wawiernia B, Busche T, Bleidt A, Horbal L, Myronovskyi M, Ahmed Y *et al*: **Characterization of Sigma Factor Genes in *Streptomyces lividans* TK24 Using a Genomic Library-Based Approach for Multiple Gene Deletions**. *Frontiers in Microbiology* 2018, **9**(3033).

10. Li A, Piel J: **A gene cluster from a marine Streptomyces encoding the biosynthesis of the aromatic spiroketal polyketide griseorhodin A**. *Chem Biol* 2002, **9**(9):1017-1026.

11. Wyszynski FJ, Lee SS, Yabe T, Wang H, Gomez-Escribano JP, Bibb MJ, Lee SJ, Davies GJ, Davis BG: **Biosynthesis of the tunicamycin antibiotics proceeds via unique exo-glycal intermediates**. *Nat Chem* 2012, **4**(7):539-546.

12. Lopatniuk M, Myronovskyi M, Luzhetskyy A: ***Streptomyces albus*: A New Cell Factory for Non-Canonical Amino Acids Incorporation into Ribosomally Synthesized Natural Products**. *Acs Chem Biol* 2017, **12**(9):2362-2370.
