# Supplementary material for: Engineering of Streptomyces lividans for heterologous expression of secondary metabolite gene clusters
Source: Microb Cell Fact. 2020 Jan 9;19:5. doi: 10.1186/s12934-020-1277-8 (PMC6950998; doi:10.1186/s12934-020-1277-8)
Supplement: Supplementary file 2 — Additional file 2: Fig. S1. BPC chromatogram extract of S. lividans TK24. [file 12934_2020_1277_MOESM2_ESM.docx]

**Additional file 2**

**Engineering of *Streptomyces lividans* for heterologous expression of secondary metabolite gene clusters**

Yousra Ahmed^1^, Yuriy Rebets^1^, Marta Rodríguez Estévez^1^, Josef Zapp^2^, Maksym Myronovskyi^1^, Andriy Luzhetskyy^1, 3,^*****

^1^Pharmazeutische Biotechnologie, Universität des Saarlandes, Saarbrücken, Germany

^2^Pharmazeutische Biologie, Universität des Saarlandes, Saarbrücken, Germany

^3^Helmholtz-Institut für Pharmazeutische Forschung Saarland, Saarbrücken, Germany

***Correspondence:** [**a.luzhetskyy@mx.uni-saarland.de**](mailto:a.luzhetskyy@mx.uni-saarland.de)**.**

A full list of author information is available at the end of the article.


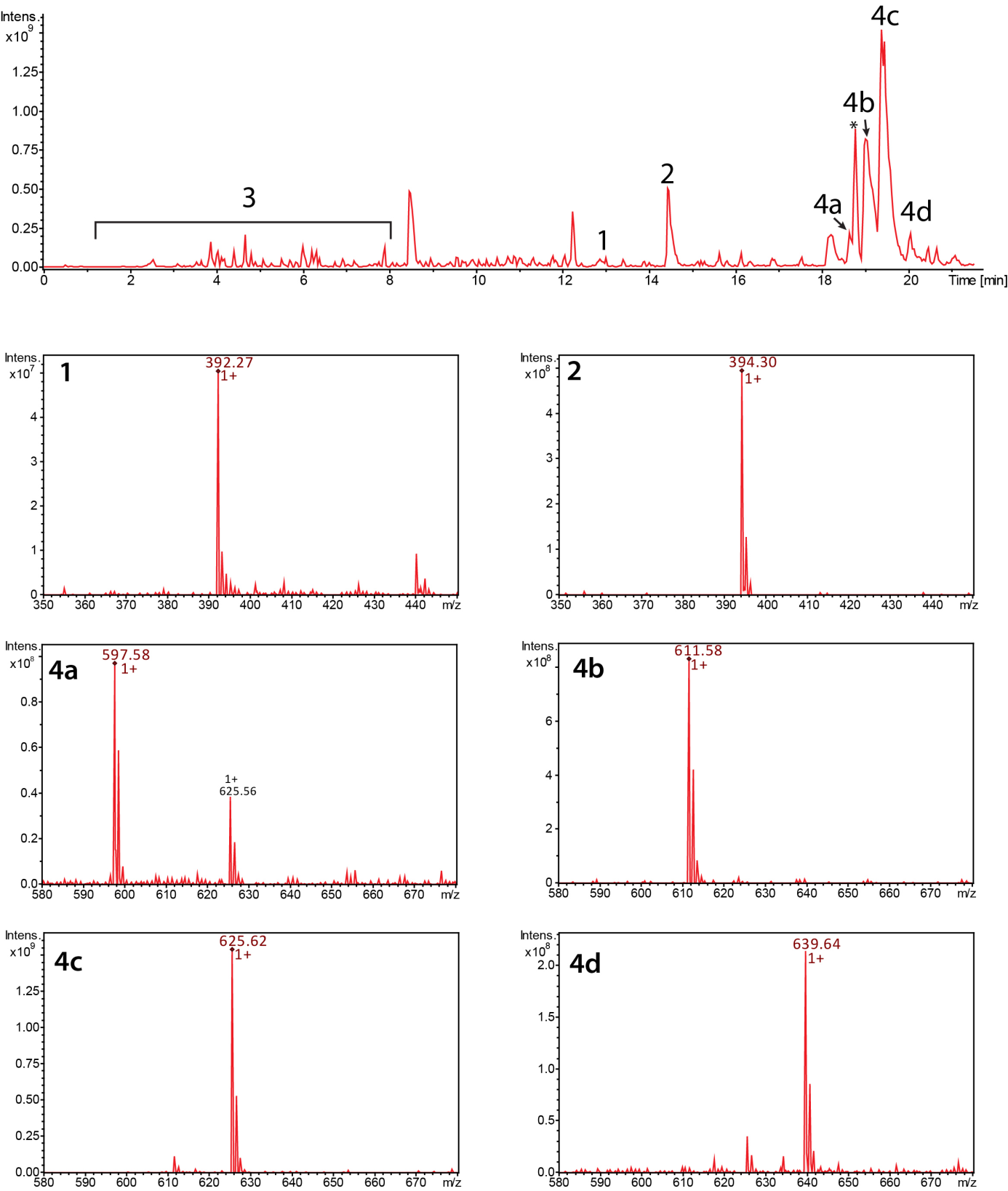


**Fig. S1**. **BPC chromatogram extract of *S. lividans* TK24.** The strain was grown in SG medium for 6 days at 28°C. The metabolites were extracted with ethyl acetate and measured on LC-MS amaZon speed system (Bruker Daltonics, Germany) with 18 min gradient protocol. Mass spectra is shown for compounds: Coelibactin (**1**), *m/z* 392.27 [*M*+H]^+^ (calculated 392.08 [*M*+H]^+^); Undecylprodigiosin (**2**), *m/z* 394.30 [*M*+H]^+^ (calculated 394.28 [*M*+H), the homologous series of 1-MG derivatives (**4a-d**), *m/z* 597.58, 611.58, 625,62, 639,64 [*M*+H]^+^, respectively.
