# Supplementary material for: Engineering of Streptomyces lividans for heterologous expression of secondary metabolite gene clusters
Source: Microb Cell Fact. 2020 Jan 9;19:5. doi: 10.1186/s12934-020-1277-8 (PMC6950998; doi:10.1186/s12934-020-1277-8)
Supplement: Supplementary file 4 — Additional file 4: Fig. S3. Comparison of S. albus subsp. chlorinus NRPS gene cluster with described PBD biosynthetic gene clusters. [file 12934_2020_1277_MOESM4_ESM.docx]

**Additional file 4**

**Engineering of *Streptomyces lividans* for heterologous expression of secondary metabolite gene clusters**

Yousra Ahmed^1^, Yuriy Rebets^1^, Marta Rodríguez Estévez^1^, Josef Zapp^2^, Maksym Myronovskyi^1^, Andriy Luzhetskyy^1, 3,^*****

^1^Pharmazeutische Biotechnologie, Universität des Saarlandes, Saarbrücken, Germany

^2^Pharmazeutische Biologie, Universität des Saarlandes, Saarbrücken, Germany

^3^Helmholtz-Institut für Pharmazeutische Forschung Saarland, Saarbrücken, Germany

***Correspondence:** [**a.luzhetskyy@mx.uni-saarland.de**](mailto:a.luzhetskyy@mx.uni-saarland.de)**.**

A full list of author information is available at the end of the article.

**
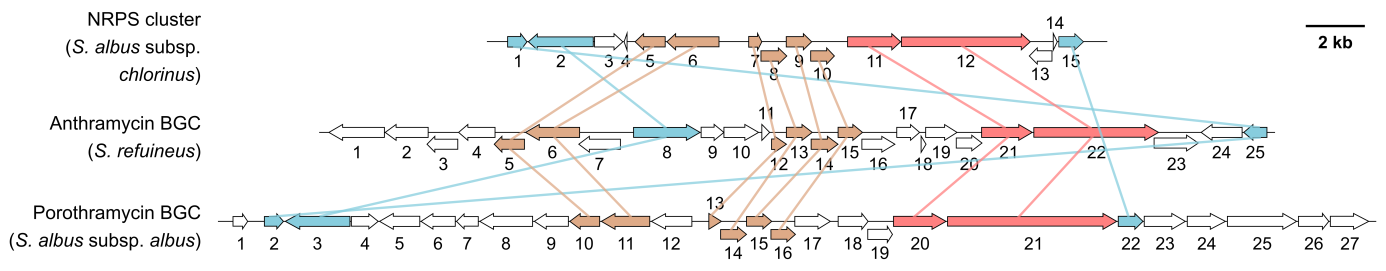
**

**Fig. S3. Comparison of *S. albus* subsp*. chlorinus* NRPS gene cluster with described PBD biosynthetic gene clusters:** anthramycin BGC from *S. refuineus* and porothramycin BGC from *S. albus* subsp. *albus*. Numbers refer to the respective genes from each cluster: in *S. albus* subsp*. chlorinus* NRPS cluster, *chl1*; in anthramycin gene cluster, *orf1*; in porothramycin gene cluster, *por1*. Genes coding for regulatory or transport proteins are coloured in blue; genes coding for APD precursor (4-alkyl-L-proline derivatives) biosynthetic enzymes are coloured in brown and genes coding for NRPSs are coloured in red. Homologous genes are connected by lines.
