# Supplementary material for: Engineering of Streptomyces lividans for heterologous expression of secondary metabolite gene clusters
Source: Microb Cell Fact. 2020 Jan 9;19:5. doi: 10.1186/s12934-020-1277-8 (PMC6950998; doi:10.1186/s12934-020-1277-8)
Supplement: Supplementary file 5 — Additional file 5: Fig. S4. Structures of several representatives of pyrrolobenzodiazepines (PBDs) family. [file 12934_2020_1277_MOESM5_ESM.docx]

**Additional file 5**

**Engineering of *Streptomyces lividans* for heterologous expression of secondary metabolite gene clusters**

Yousra Ahmed^1^, Yuriy Rebets^1^, Marta Rodríguez Estévez^1^, Josef Zapp^2^, Maksym Myronovskyi^1^, Andriy Luzhetskyy^1, 3,^*****

^1^Pharmazeutische Biotechnologie, Universität des Saarlandes, Saarbrücken, Germany

^2^Pharmazeutische Biologie, Universität des Saarlandes, Saarbrücken, Germany

^3^Helmholtz-Institut für Pharmazeutische Forschung Saarland, Saarbrücken, Germany

***Correspondence:** [**a.luzhetskyy@mx.uni-saarland.de**](mailto:a.luzhetskyy@mx.uni-saarland.de)**.**

A full list of author information is available at the end of the article.


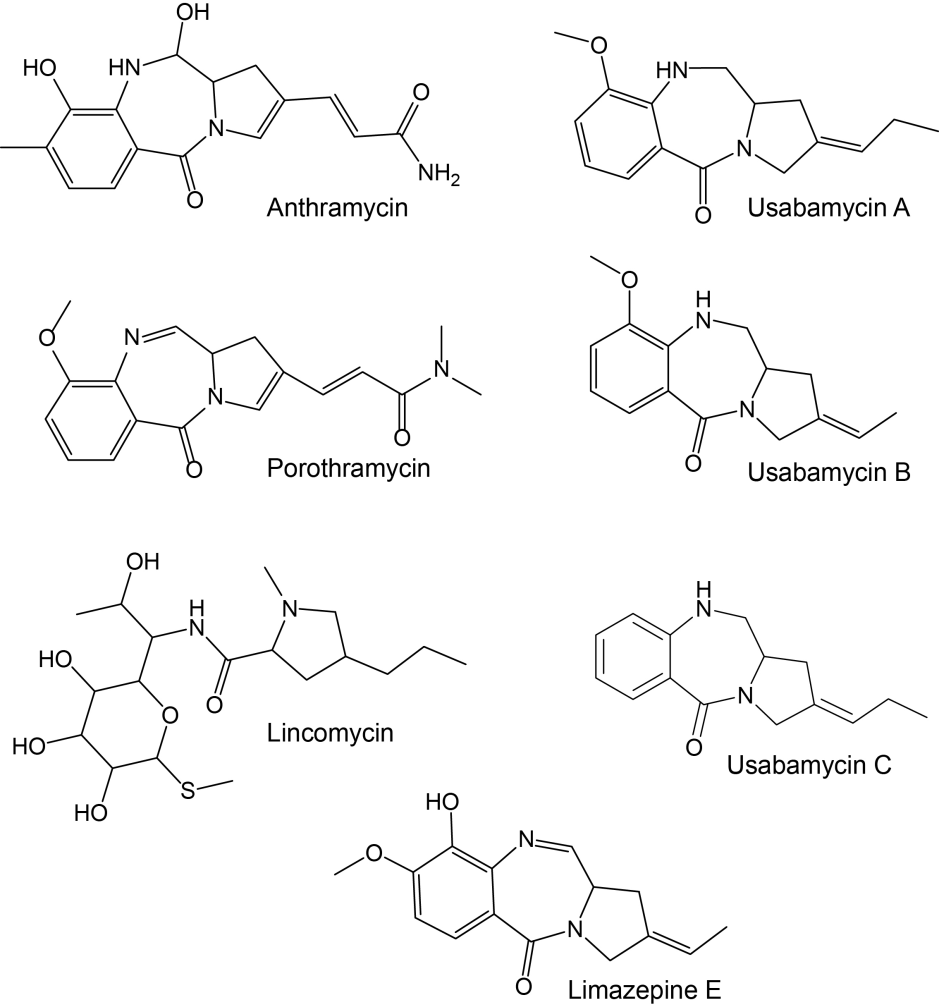


**Fig. S4. Structures of several representatives of of pyrrolobenzodiazepines (PBDs) family**.
