# Supplementary material for: Engineering of Streptomyces lividans for heterologous expression of secondary metabolite gene clusters
Source: Microb Cell Fact. 2020 Jan 9;19:5. doi: 10.1186/s12934-020-1277-8 (PMC6950998; doi:10.1186/s12934-020-1277-8)
Supplement: Supplementary file 6 — Additional file 6: Table S2. Homologous genes in described PBD biosynthetic gene clusters. [file 12934_2020_1277_MOESM6_ESM.docx]

**Additional file 6**

**Engineering of *Streptomyces lividans* for heterologous expression of secondary metabolite gene clusters**

Yousra Ahmed^1^, Yuriy Rebets^1^, Marta Rodríguez Estévez^1^, Josef Zapp^2^, Maksym Myronovskyi^1^, Andriy Luzhetskyy^1, 3,^*****

^1^Pharmazeutische Biotechnologie, Universität des Saarlandes, Saarbrücken, Germany

^2^Pharmazeutische Biologie, Universität des Saarlandes, Saarbrücken, Germany

^3^Helmholtz-Institut für Pharmazeutische Forschung Saarland, Saarbrücken, Germany

***Correspondence:** [**a.luzhetskyy@mx.uni-saarland.de**](mailto:a.luzhetskyy@mx.uni-saarland.de)**.**

A full list of author information is available at the end of the article.

**Table S2. Homologous genes in described PBD biosynthetic gene clusters**

| *Streptomyces* *albus* subsp. *chlorinus* NRPS cluster gene | Predicted function | Homologue in anthramycin BGC | Identity (%) | Homologue in porothramycin BGC | Identity (%) |
| --- | --- | --- | --- | --- | --- |
| *chl1* | Transcriptional regulatory protein | *orf25* | 67 | *por2* | 91 |
| *chl2* | UvrABC system protein | *orf8* | 49 | *por3* | 93 |
| *chl3* | HTH-domain protein | - | - | *por4* | 92 |
| *chl4* | YafY family transcriptional regulator | - | - | - | - |
| *chl5* | Putative methyltransferase | *orf5* | 76 | *por10* | 90 |
| *chl6* | Gamma-glutamyltransferase | *orf6* | 80 | *por11* | 92 |
| *chl7* | Putative L-DOPA 2,3-dioxygenase | *orf12* | 74 | *por13* | 89 |
| *chl8* | Putative tyrosine hydroxylase | *orf13* | 58 | *por14* | 83 |
| *chl9* | Putative F-420 dependent reductase | *orf14* | 82 | *por15* | 91 |
| *chl10* | Putative isomerase | *orf15* | 76 | *por16* | 86 |
| *chl11* | Putative NRPS | *orf21* | 72 | *por20* | 87 |
| *chl12* | Putative NRPS + kinurenine monooxygenase | *orf22* | 71 | *por21* | 89 |
| *chl13* | Hypothetical protein | - | - | - | - |
| *chl14* | Hypothetical protein | - | - | - | - |
| *chl15* | NmrA family transcriptional regulator | - | - | por22 | 90 |
