# Supplementary material for: Engineering of Streptomyces lividans for heterologous expression of secondary metabolite gene clusters
Source: Microb Cell Fact. 2020 Jan 9;19:5. doi: 10.1186/s12934-020-1277-8 (PMC6950998; doi:10.1186/s12934-020-1277-8)
Supplement: Supplementary file 7 — Additional file 7: Fig. S5. Fragmentation pattern of compounds identified in the extract of S. lividans ΔYA9 with BAC2I4. [file 12934_2020_1277_MOESM7_ESM.docx]

**Additional file 7**

**Engineering of *Streptomyces lividans* for heterologous expression of secondary metabolite gene clusters**

Yousra Ahmed^1^, Yuriy Rebets^1^, Marta Rodríguez Estévez^1^, Josef Zapp^2^, Maksym Myronovskyi^1^, Andriy Luzhetskyy^1, 3,^*****

^1^Pharmazeutische Biotechnologie, Universität des Saarlandes, Saarbrücken, Germany

^2^Pharmazeutische Biologie, Universität des Saarlandes, Saarbrücken, Germany

^3^Helmholtz-Institut für Pharmazeutische Forschung Saarland, Saarbrücken, Germany

***Correspondence:** [**a.luzhetskyy@mx.uni-saarland.de**](mailto:a.luzhetskyy@mx.uni-saarland.de)**.**

A full list of author information is available at the end of the article.


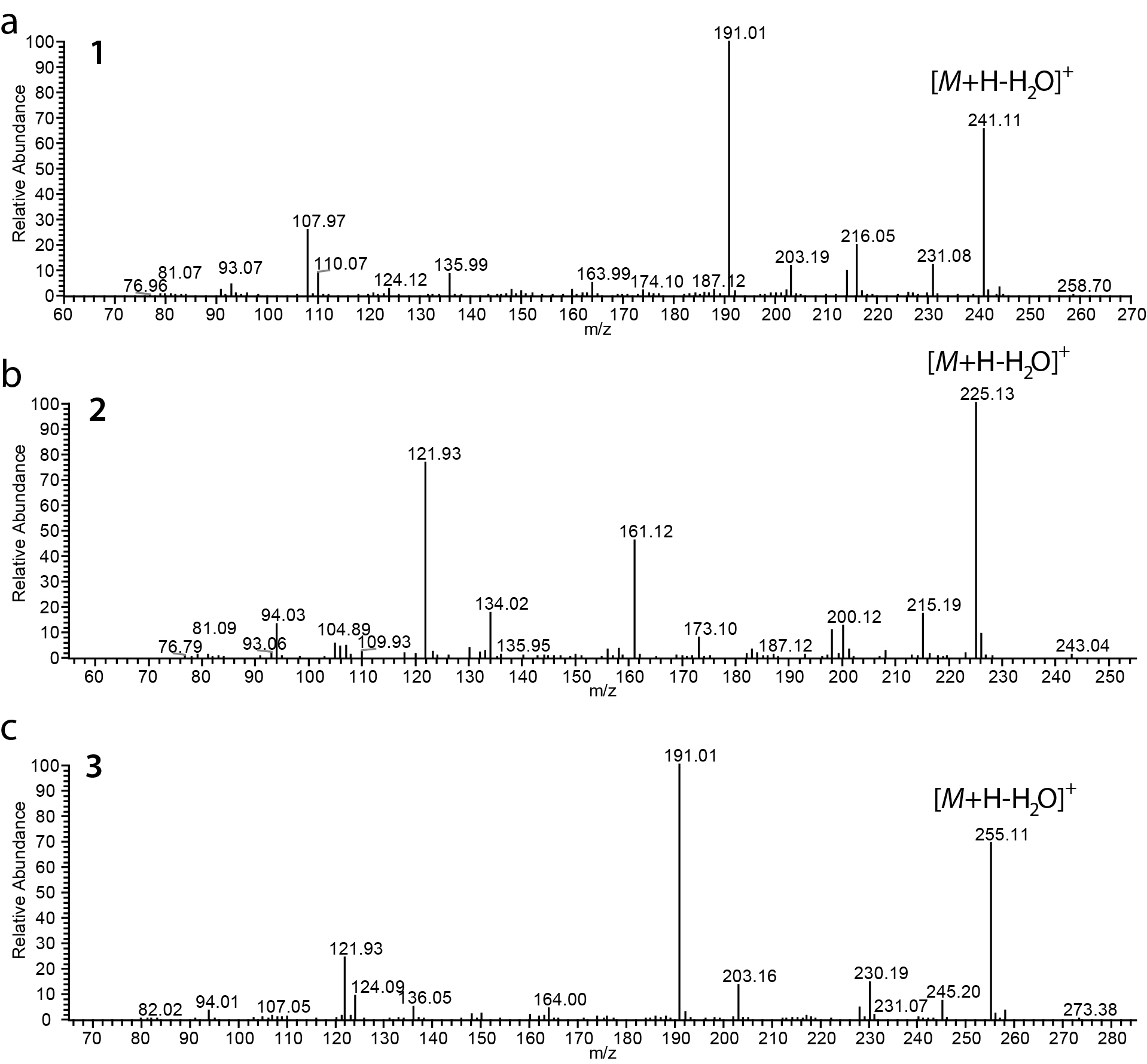


**Fig. S5. Fragmentation pattern of compounds indetified in the extract of *S. lividans* ΔYA9 with BAC2I4**. Mass chromatogram of metabolites of *S. lividans* ΔYA9 with BAC2I4 grown in DNPM medium for 6 days at 28°C. The metabolites were extracted with ethyl acetate and the accurate mass was determined using Thermo LTQ Orbitrap XL coupled to UPLC Thermo Dionex Ultimate 3000 RS with 20 min gradient protocol. **(a)** Fragmentation pattern of compound **1** with *m/z* 259.14377 [*M*+H]^+^, **(b)** fragmentation pattern of compound **2** with *m/z* 243.14883 [*M*+H]^+^, and **(c)** fragmentation pattern of compound **3** with *m/z* 273.15933 [*M*+H]^+^.
