# Supplementary material for: Engineering of Streptomyces lividans for heterologous expression of secondary metabolite gene clusters
Source: Microb Cell Fact. 2020 Jan 9;19:5. doi: 10.1186/s12934-020-1277-8 (PMC6950998; doi:10.1186/s12934-020-1277-8)
Supplement: Supplementary file 8 — Additional file 8: Table S3. Strains, Plasmids and BACs used in this study. [file 12934_2020_1277_MOESM8_ESM.docx]

**Additional file 8**

**Engineering of *Streptomyces lividans* for heterologous expression of secondary metabolite gene clusters**

Yousra Ahmed^1^, Yuriy Rebets^1^, Marta Rodríguez Estévez^1^, Josef Zapp^2^, Maksym Myronovskyi^1^, Andriy Luzhetskyy^1, 3,^*****

^1^Pharmazeutische Biotechnologie, Universität des Saarlandes, Saarbrücken, Germany

^2^Pharmazeutische Biologie, Universität des Saarlandes, Saarbrücken, Germany

^3^Helmholtz-Institut für Pharmazeutische Forschung Saarland, Saarbrücken, Germany

***Correspondence:** [**a.luzhetskyy@mx.uni-saarland.de**](mailto:a.luzhetskyy@mx.uni-saarland.de)**.**

A full list of author information is available at the end of the article.

**Table. S3. Strains, Plasmids and BACs used in this study**

| **Strains** | **Features** | **Reference or source** |
| --- | --- | --- |
| ***Streptomyces strains*** | | |
| *Streptomyces lividans* TK24 | *S. lividans* wild type | [[2](#_ENREF_2)] |
| *Streptomyces albus* Del14 | Derivative of S. albus J1074 with deletion of 15 gene clusters | [[3](#_ENREF_3)] |
| *Streptomyces coelicolor* M1154 | Derivative of *S. coelicolor* M1152 with *rpsL* mutation | [[4](#_ENREF_4)] |
| *Streptomyces lividans* ΔYA9 | Derivative of *S. lividans* TK24 with 9 inactivated gene clusters | This work |
| *Streptomyces lividans Δ*YA10 | Derivative of *S. lividans* ΔYA9 with 10 inactivated gene clusters and one additional *attB* site | This work |
| *Streptomyces lividans* ΔYA11 | Derivative of *S. lividans* ΔYA10 with 11 inactivated gene clusters and two additional *attB* site | This work |
| ***E. coli strains*** | | |
| *E. coli* ET12567 pUB307 | Donor strain for intergeneric conjugation | [[5](#_ENREF_5)] |
| *E. coli* WM6026 | Donor strain for intergeneric conjugation | [[6](#_ENREF_6)] |
| *E. coli* GB05-red | Strain used for Red/ET | [[7](#_ENREF_7)] |
| **Plasmids** | | |
| patt-saac-oriT | Resistance cassette plasmid containing a synthetic fragment with *aac(3)IV*,  *oriT*, *B-CC*, *P-GG* and *loxP* sites | [[8](#_ENREF_8)] |
| pUWLint31 | pUWLCREdeltaKpnI with the *XbaI/BamHI* fragment of pKHint31 containing *int* | [[8](#_ENREF_8)] |
| phygattB | Resistance cassette plasmid containing hygromycin resistance marker flanked with *MssI* restriction sites and *attB* sequence | [[3](#_ENREF_3)] |
| **BACs** | | |
| pSMARTgus | Derivative of pSMART with the *gusA* gene | [[8](#_ENREF_8)] |
| 1629 | pSMARTgus derivative containing a fragment of the *S. lividans* chromosome with Undecylprodigiosin cluster №10 | [[9](#_ENREF_9)] |
| 1704 | pSMARTgus derivative containing a fragment of the *S. lividans* chromosome with Actinorhodin №14 | [[9](#_ENREF_9)] |
| 1443 | pSMARTgus derivative containing a fragment of the *S. lividans* chromosome with melanin cluster №17 | [[9](#_ENREF_9)] |
| 1320 | pSMARTgus derivative containing a fragment of the *S. lividans* chromosome with melanin cluster №13 | [[9](#_ENREF_9)] |
| 1715 | pSMARTgus derivative containing a fragment of the *S. lividans* chromosome with germicidin cluster №19 | [[9](#_ENREF_9)] |
| 1008 | pSMARTgus derivative containing a fragment of the *S. lividans* chromosome with CDAcluster №15 | [[9](#_ENREF_9)] |
| 1161 | pSMARTgus derivative containing a fragment of the *S. lividans* chromosome with coelimycin cluster №6 | [[9](#_ENREF_9)] |
| 1250 | pSMARTgus derivative containing a fragment of the *S. lividans* chromosome with coelibactin cluster №2 | [[9](#_ENREF_9)] |
| 490 | pSMARTgus derivative containing a fragment of the *S. lividans* chromosome with t1PKS cluster №24 | [[9](#_ENREF_9)] |
| 1468 | pSMARTgus derivative containing a fragment of the *S. lividans* chromosome with coelichelin cluster №21 | [[9](#_ENREF_9)] |
| 1092 | pSMARTgus derivative containing a fragment of the *S. lividans* chromosome with abCPK cluster №5 | [[9](#_ENREF_9)] |
| 1629-am | Derivative of 1629 with №10 gene cluster fragment substituted with a cassette from patt-saac-oriT | This work |
| 1704-am | Derivative of 1704 with №14 gene cluster fragment substituted with a cassette from patt-saac-oriT | This work |
| 1443-am | Derivative of 1443 with №17 gene cluster fragment substituted with a cassette from patt-saac-oriT | This work |
| 1320-am | Derivative of 1320 with №13 gene cluster fragment substituted with a cassette from patt-saac-oriT | This work |
| 1715-am | Derivative of 1715 with №19 gene cluster fragment substituted with a cassette from patt-saac-oriT | This work |
| 1008-am | Derivative of 1008 with №15 gene cluster fragment substituted with a cassette from patt-saac-oriT | This work |
| **Table. S3 (continued)** | | |
| 1161-am | Derivative of 1161 with№6 gene cluster fragment substituted with a cassette from patt-saac-oriT | This work |
| 1250-am | Derivative of 1250 with№2 gene cluster fragment substituted with a cassette from patt-saac-oriT | This work |
| 490-am | Derivative of 490 with №24 gene cluster fragment substituted with a cassette from patt-saac-oriT | This work |
| 1468::hygattB | Derivative of 1468 with №24 gene cluster fragment substituted with a cassette from phygattB | This work |
| 1092::hygattB | Derivative of 1092 with №5 gene cluster fragment substituted with a cassette from phygattB | This work |
| 1468::attB | Derivative of 1468::hygattB after cutting out the *hyg* gene with *MssI* | This work |
| 1092::attB | Derivative of 1092::hygattB after cutting out the *hyg* gene with *MssI* | This work |
| 1468::attBamoriT | Derivative of 1468::attB with substitution of *cat* gene with *aac(3)IV*-OriT fragment | This work |
| 1092::attBamoriT | Derivative of 1092::attB with substitution of *cat* gene with *aac(3)IV*-OriT fragment | This work |
| 2I4 | pSMART derivative containing a fragment from *S. albus* subsp*. chlorinus* NRRL B-24108 with NRPS gene cluster | Intact Genomics, USA |
| PMP31 | pOJ436 derivative, containing the griseorhodin biosynthetic cluster | [[10](#_ENREF_10)] |
| pIJ12003a | 12.9 Kbp *tun-*gene cluster cloned into the pRT802 | [[11](#_ENREF_11)] |
| pCindelX | Plasmid containing cinnamycin with deletion of OH group gene cluster and *aac(3)IV* resistance marker | [[12](#_ENREF_12)] |

**References**

1. Busche T, Tsolis KC, Koepff J, Rebets Y, Ruckert C, Hamed MB, Bleidt A, Wiechert W, Lopatniuk M, Yousra A *et al*: **Multi-Omics and Targeted Approaches to Determine the Role of Cellular Proteases in *Streptomyces* Protein Secretion**. *Front Microbiol* 2018, **9**:1174.

2. Ruckert C, Albersmeier A, Busche T, Jaenicke S, Winkler A, Friethjonsson OH, Hreggviethsson GO, Lambert C, Badcock D, Bernaerts K *et al*: **Complete genome sequence of *Streptomyces lividans* TK24**. *J Biotechnol* 2015, **199**:21-22.

3. Myronovskyi M, Rosenkranzer B, Nadmid S, Pujic P, Normand P, Luzhetskyy A: **Generation of a cluster-free *Streptomyces albus* chassis strains for improved heterologous expression of secondary metabolite clusters**. *Metab Eng* 2018.

4. Gomez-Escribano JP, Bibb MJ: **Engineering *Streptomyces coelicolor* for heterologous expression of secondary metabolite gene clusters**. *Microb Biotechnol* 2011, **4**(2):207-215.

5. Flett F, Mersinias V, Smith CP: **High efficiency intergeneric conjugal transfer of plasmid DNA from Escherichia coli to methyl DNA-restricting streptomycetes**. *Fems Microbiol Lett* 1997, **155**(2):223-229.

6. Blodgett JA, Thomas PM, Li G, Velasquez JE, van der Donk WA, Kelleher NL, Metcalf WW: **Unusual transformations in the biosynthesis of the antibiotic phosphinothricin tripeptide**. *Nat Chem Biol* 2007, **3**(8):480-485.

7. Fu J, Teucher M, Anastassiadis K, Skarnes W, Stewart AF: **A Recombineering Pipeline to Make Conditional Targeting Constructs**. *Method Enzymol* 2010, **477**:125-144.

8. Myronovskyi M, Rosenkranzer B, Luzhetskyy A: **Iterative marker excision system**. *Appl Microbiol Biotechnol* 2014, **98**(10):4557-4570.

9. Rebets Y, Tsolis KC, Guðmundsdóttir EE, Koepff J, Wawiernia B, Busche T, Bleidt A, Horbal L, Myronovskyi M, Ahmed Y *et al*: **Characterization of Sigma Factor Genes in *Streptomyces lividans* TK24 Using a Genomic Library-Based Approach for Multiple Gene Deletions**. *Frontiers in Microbiology* 2018, **9**(3033).

10. Li A, Piel J: **A gene cluster from a marine Streptomyces encoding the biosynthesis of the aromatic spiroketal polyketide griseorhodin A**. *Chem Biol* 2002, **9**(9):1017-1026.

11. Wyszynski FJ, Lee SS, Yabe T, Wang H, Gomez-Escribano JP, Bibb MJ, Lee SJ, Davies GJ, Davis BG: **Biosynthesis of the tunicamycin antibiotics proceeds via unique exo-glycal intermediates**. *Nat Chem* 2012, **4**(7):539-546.

12. Lopatniuk M, Myronovskyi M, Luzhetskyy A: ***Streptomyces albus*: A New Cell Factory for Non-Canonical Amino Acids Incorporation into Ribosomally Synthesized Natural Products**. *Acs Chem Biol* 2017, **12**(9):2362-2370.
