# Supplementary material for: Engineering of Streptomyces lividans for heterologous expression of secondary metabolite gene clusters
Source: Microb Cell Fact. 2020 Jan 9;19:5. doi: 10.1186/s12934-020-1277-8 (PMC6950998; doi:10.1186/s12934-020-1277-8)
Supplement: Supplementary file 9 — Additional file 9: Table S4. Primers used in this study. [file 12934_2020_1277_MOESM9_ESM.docx]

**Additional file 9**

**Engineering of *Streptomyces lividans* for heterologous expression of secondary metabolite gene clusters**

Yousra Ahmed^1^, Yuriy Rebets^1^, Marta Rodríguez Estévez^1^, Josef Zapp^2^, Maksym Myronovskyi^1^, Andriy Luzhetskyy^1, 3,^*****

^1^Pharmazeutische Biotechnologie, Universität des Saarlandes, Saarbrücken, Germany

^2^Pharmazeutische Biologie, Universität des Saarlandes, Saarbrücken, Germany

^3^Helmholtz-Institut für Pharmazeutische Forschung Saarland, Saarbrücken, Germany

***Correspondence:** [**a.luzhetskyy@mx.uni-saarland.de**](mailto:a.luzhetskyy@mx.uni-saarland.de)**.**

A full list of author information is available at the end of the article.

**Table. S4. Primers used in this study**

| **Primers** | **Sequences** | **Description** |
| --- | --- | --- |
| Cl10_F | CTACGTGGACGAGGAGGAGTCCTTCGTACGGTCACGGTCCTGGTGGTGGTTCGACCCGGTACCGGAGTA | Deletion of cluster Nr° 10 |
| Cl10_R | CAACGCGCGACATGGCGGGGCAGCAGGAACGGATACGGGCGGCGCAGGATACTACGCCCCCAACTGAGAG |  |
| Cl13_F | GTGAACGACGGCGTGAACGGCGTCGCACTCGCCGTCTTCATCTTCTTCTTTCGACCCGGTACCGGAGTA | Deletion of cluster Nr° 13 |
| Cl13_R | GTGAGGATCTTCGGCAAGGCACGGCACCGGCCCTCCGCCTCTTGGCGGCAACTACGCCCCCAACTGAGAG |  |
| Cl14_F | GAGCCCCCGAACACTCCCGGCCACGGCCCTGCTTGAGGGGCTGCCCGACGTCGACCCGGTACCGGAGTA | Deletion of cluster Nr° 14 |
| Cl14_R | TGGTCGCGGATCGCCCGGCTGGTGTCCGGCCGTCCGCGTGCCGTCTGGGTACTACGCCCCCAACTGAGAG |  |
| Cl17_F | TCCACCGGGTCGCCGACTCGTGCTCGTCGGCCGGGCGGTGGGTGCGGAACTCGACCCGGTACCGGAGTA | Deletion of cluster Nr° 17 |
| Cl17_R | GAGGCGCTGCTCGCCGCGCTGCACGAGGGCCGCATCGCGGGTGCGGGCGTACTACGCCCCCAACTGAGAG |  |
| Cl19_F | TGAGGTCAACTCCGCCGGGCGGCTGCCGTACGGGCAGCTTTCCGGGGATCCGTCGACCC | Deletion of cluster Nr° 19 |
| Cl19_R | ACTGGTACCTGACGAGCGTTCAGGCGGGGTTCGAGCCGTTGTAGGCTGGAGCTGCTTCG |  |
| Cl15_F | TAGTGCGGGTCGATCGGCAGGTAGGCGGCCCCGGACTTGTTCCGGGGATCCGTCGACCC | Deletion of cluster Nr° 15 |
| Cl15_R | GTGTCTGAGAACTCTTCGGTTCGGCACGGTCTGACGAGCTGTAGGCTGGAGCTGCTTCG |  |
| Cl6_F | TCCCTTCAGGCGCTGGTGACGGGCTCTTCGCGGGCCGCGTTCCGGGGATCCGTCGACCC | Deletion of cluster Nr° 6 |
| Cl6_R | ATGGTGTGGTGGAGGGGTTGGTCGAGGTGGGGGTCGAGTTGTAGGCTGGAGCTGCTTCG |  |
| Cl2_F | AGGAACTCGATGGTCCCGTCGGGCAGGTAGCGGCCGAGGTTCCGGGGATCCGTCGACCC | Deletion of cluster Nr° 2 |
| Cl2_R | TGGTACGGCGAGAAGGAAGTCACCGCGGTCGCCCAGGCGTGTAGGCTGGAGCTGCTTCG |  |
| Cl24_F1 | AACGCATGCGGGTCTCACTTCACGGCGGGTTCGGACAGGTTCCGGGGATCCGTCGACCC | Deletion of cluster Nr° 24 |
| Cl24_R | AAACGGCAGCCTGGAGAGCGCCCCCTGCGGTGTCAACCTTGTAGGCTGGAGCTGCTTCG |  |
| Cl21attB_F | CGTGGTCGAAGATCATGAACCGGGTGCTGGTGGTTCCCTTGTAGGCTGGAGCTGCTTCG | Deletion of cluster Nr° 21 |
| Cl21attB_R | AACAGGCCGGTGCGACCGTGCGGGTCTCCAGGTCGGCCTTTCCGGGGATCCGTCGACCC |  |
| Cl5attB_F | GGCGTGTTCAGGGCCGGCGGCCGTCCGGGATCAGGACGGTGTAGGCTGGAGCTGCTTCG | Deletion of cluster Nr° 5 |
| Cl5attB_R | ACGCATGTCGTTCCTGCATGACCTGCTCACGGCGCAGGCTTCCGGGGATCCGTCGACCC |  |
| Cm_F | TTCAGGAGCTAAGGAAGCTAAAATGGAGAAAAAAATCACTTCCGGGGATCCGTCGACCC | Substitution of *cat* gene with *aac (3)IV* gene and *oriT* sequence |
| Cm_R | AGGCTTTTGACTTCTGTCACCTAGGTTACGCCCCGCCCTTGTAGGCTGGAGCTGCTTCG |  |
| Cl10C_F | GCGAGACCGGCTCCGAGA | Verification cluster Nr° 10 deletion |
| Cl10C_R | GTGCGTGATCCACAGCGGA |  |
| Cl13C_F | TCGGCATCCCGTTCTTCTA | Verification cluster Nr° 13 deletion |
| Cl13C_R | TCCCGAGGCAGCCTTACCGA |  |
| Cl14C_F | CTGGGGGAACGACCGGATT | Verification cluster Nr° 14 deletion |
| Cl14C_R | TGGTCGGCCTGCTGTCGAT |  |
| Cl17C_F | TCGGGACGCGGGCCGAC | Verification cluster Nr° 17 deletion |
| Cl17C_R | CTCCGTGCACCTGGCGCT |  |
| Cl19C_F | AGTTGCTGCACGGTCTCAC | Verification cluster Nr° 19 deletion |
| Cl19C_R | AACGGCTCGAACGACGTG |  |
| Cl15C_F | AGGTGTAGATCGTGTACGC | Verification cluster Nr° 15deletion |
| Cl15C_R | AATAAGCATATTGACCAGCT |  |
| Cl6C_F | AAGTCGAGTACGGCGATCT | Verification cluster Nr° 6 deletion |
| Cl6C_R | TGAGTTCGCCGATGGAGTGT |  |
| Cl2C_F | CGAGTCGTCCACGACGA | Verification cluster Nr° 2deletion |
| Cl2C_R | GGACCTCTTCGCCGCGT |  |
| Cl24C_F1 | CCGCCGTCAGACTCGTA | Verification cluster Nr° 24 deletion |
| Cl24C_R | GCGTGCTGTCGGTAGGAA |  |
| Cl21C_F | TTGCGCAGCGCGTTCTGCAT | Verification cluster Nr° 21 deletion |
| Cl21C_R | TGTCGCGTGTCGCCTGTCA |  |
| Cl5C_F | CACGCCGTCCGTACGAGT | Verification cluster Nr° 5 deletion |
| Cl5C_R | AACCGGCGCTCACCGGC |  |
| CmC_F | AATCTGATCGGCACGTAAGA | Verification Substitution of *cat* gene |
| CmC_R | GTCCGTGGAATGAACAATG |  |
